# Cgm1 is a β-galactoside α-(1 → 4)-mannosyltransferase involved in the biosynthesis of capsular glucuronoxylomannogalactan in *Cryptococcus neoformans*

**DOI:** 10.1016/j.jbc.2025.110632

**Published:** 2025-08-26

**Authors:** Chihiro Kadooka, Yutaka Tanaka, Ko Sato, Hayato Sato, Daisuke Hira, Shun Yakabe, Kazuyoshi Kawakami, Takuji Oka

**Affiliations:** 1 Department of Biotechnology and Life Sciences, Faculty of Biotechnology and Life Sciences, Sojo University, Kumamoto, Japan; 2 Division of Infection and Host Defense, Tohoku Medical and Pharmaceutical University, Sendai, Japan; 3 Department of Clinical Microbiology and Infection, Tohoku University Graduate School of Medicine, Sendai, Miyagi, Japan; 4 Department of Plastic and Reconstructive Surgery, Tohoku University Graduate School of Medicine, Sendai, Miyagi, Japan; 5 Department of Medical Microbiology, Mycology and Immunology, Tohoku University Graduate School of Medicine, Sendai, Miyagi, Japan; 6 Division of Internal Medicine, Hirose Hospital, Sendai, Miyagi, Japan

**Keywords:** mannosyltransferase, glucronoxylomannogalactan, *Cryptococcus neoformans*, virulence factor, immune evasion

## Abstract

Capsular polysaccharides are present in the outermost layer of the cell wall of *Cryptococcus neoformans*. The capsule consists of glucuronoxylomannan and glucuronoxylomannogalactan (GXMGal), both of which are major virulence factors that enable immune evasion. This study aimed to identify a novel glycosyltransferase involved in the biosynthesis of capsular polysaccharides in *C. neoformans*. While glucuronoxylomannan is the predominant capsule component and plays a broad role in immune evasion, GXMGal, despite its lower abundance, is thought to contribute to pathogenicity through its structurally unique galactomannan side chain. Glycosyltransferases involved in GXMGal biosynthesis have attracted much attention as potential targets for antifungal drug development because of their role in pathogenicity. In this study, we identified a novel β-galactoside α-(1 → 4)-mannosyltransferase, cryptococcal β-galactoside mannosyltransferase 1 (Cgm1) (glucan organizing enzyme 1 [Goe1]), which is involved in the biosynthesis of the galactomannan side chain of GXMGal. The GXMGal galactomannan side chain was almost completely lost in the *cgm1* (*goe1*) disruptant, indicating that Cgm1 (Goe1) is the α-(1 → 4)-mannosyltransferase responsible for its biosynthesis. The *cgm1* (*goe1*) disruptant exhibited temperature sensitivity at 37 °C. In addition, interferon-γ production was significantly increased in mice infected with the *cgm1* (*goe1*) disruptant, demonstrating the importance of the galactomannan side chain of GXMGal in immune evasion.

As a basidiomycetous pathogenic yeast, *Cryptococcus neoforman*s is the major cause of cryptococcosis in immunocompromised individuals, particularly those with HIV, making up most of the infected individuals. In immunocompromised individuals, the pulmonary infection elicited by *C. neoformans* can spread to the patient’s central nervous system, leading to severe meningoencephalitis, which is characterized by a high mortality rate. Cryptococcosis accounts for approximately 1,000,000 infections and 600,000 deaths annually (1). Accordingly, the World Health Organization classified *C. neoformans* as a priority fungal pathogen (https://www.who.int/publications-detail-redirect/9789240060241).

The capsular polysaccharides existing in the outermost layer of the cell wall of *C. neoformans* mediate its virulence, having multiple effects on the host’s immune system. Glucuronoxylomannan (GXM), glucuronoxylomannogalactan (GXMGal), and mannoproteins constitute 88%, 10%, and 2%, respectively, of the capsule structure (2, 3). The complexity of capsular biosynthesis is evidenced by the numerous acapsular mutants identified through screening based on colony morphology (4). Subsequent cloning of *CAP* genes, which complement these mutant phenotypes, confirmed their essential role in virulence (5, 6, 7, 8).

As a large polysaccharide, GXM has a molecular weight of 1,000,000 to 7,000,000, constituting an α-mannan core with α-(1 → 3)-linked mannose (Man), β-(1 → 2)-linked glucuronic acid (GlcA), and β-(1 → 2)- or β-(1 → 4)-linked xylose (Xyl) residues as side chains (9). In contrast, GXMGal is a smaller polysaccharide (≤100,000 molecular weight) with an α-galactan core composed of α-(1 → 6)-linked galactose (Gal) and galactomannan side chains (2). Galactofuranose (Gal*f*) residues are added to the α-galactan main chain (10), whereas Xyl and GlcA residues modify the galactomannan side chain (3). Notably, the GXM and GXMGal Man residues undergo O-acetylation at the O-2 or O-6 position, a modification linked to the host immune responses (11, 12, 13).

While *CAP* genes are essential for GXM biosynthesis and virulence, the role of GXMGal in pathogenicity has attracted much attention. Mutants lacking *UGT1* (encoding a UDP-Gal transporter) and *UGE1* (encoding UDP-Gal 4-epimerase) exhibit attenuated virulence, suggesting the significance of GXMGal in pathogenicity (14, 15, 16). The only known enzymes involved in GXMGal biosynthesis are the α-mannoside β-(1 → 2)-xylosyltransferases, Cxt1 and Cxt2 (17, 18, 19, 20), and the putative α-galactoside β-(1 → 3)-galactosyltransferase, Ggt2 (21) (Fig. 1). Disruption of *cxt1* reduces virulence in mice (18), further supporting the importance of the galactomannan side chain of GXMGal in immune evasion. Thus, it is important to understand the mechanism of GXMGal biosynthesis. The galactomannan side chain of GXMGal contains a unique α-Man residue attached to the C-4 position of the β-Gal residue, a structure not found in any other organism. Consequently, the glycosyltransferases catalyzing this reaction are expected to differ structurally from known mannosyltransferases. The Carbohydrate-Active enZymes (CAZy) database (https://www.cazy.org/) lists four putative glycosyltransferases conserved in *C. neoformans* var. *grubii* H99 (CNAG_00129, CNAG_01513, CNAG_02199, and CNAG_04449), classified as GTnc. Among them, CNAG_04449, named Hoc4 because of its homology with α-(1 → 6)-mannosyltransferases, Hoc1 and Hoc3, involved in *O*-glycan biosynthesis, has an unclear function (22). Recently, CNAG_02199, named Goe1 (glucan organizing enzyme 1), has been reported as a factor involved in the formation of β-(1 → 3)-glucan and α-(1 → 4)-glucan in the cell wall, but its detailed function remains unclear (23).

**Figure 1:**
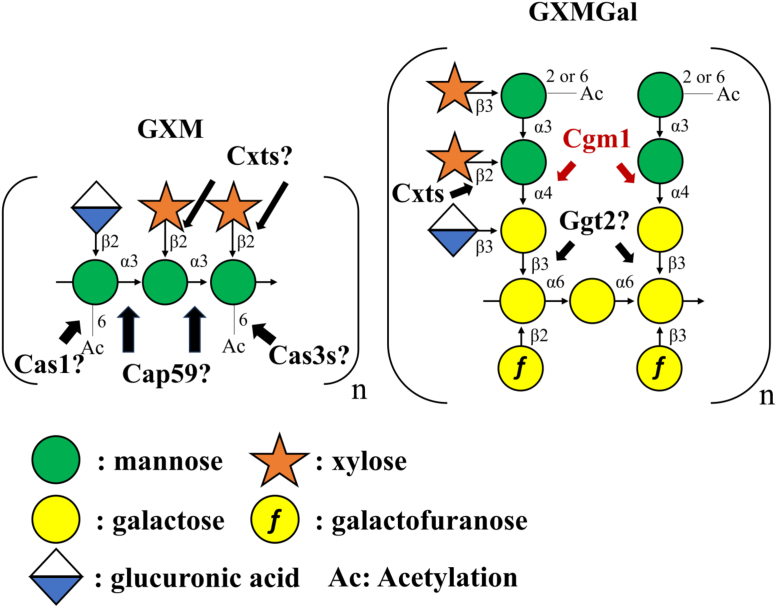
**Structural model and biosynthesis of GXM serotype A and GXMGal.** Structure of GXM serotype A and GXMGal. Cap59 represents putative GXM α-mannoside α-(1 → 3)-mannosyltransferase. Cas1 and Cas3 represent putative GXM *O*-acetyltransferases Cas1, Cas3, Cas31, Cas32, Cas33, Cas34, and Cas35. Cxts represents GXM and GXMGal α-mannoside β-(1 → 2)-xylosyltransferases Cxt1 and Cxt2. Ggt2 represents the putative GXMGal α-galactoside β-(1 → 3)-galactosyltransferase. Cgm1 represents GXMGal β-galactoside α-(1 → 4)-mannosyltransferase. GXM, glucuronoxylomannan; GXMGal, glucuronoxylomannogalactan.

In this study, cryptococcal β-galactoside mannosyltransferase 1 (Cgm1) (Goe1) (CNAG_02199) and Cgm2 (CNAG_00129), novel β-galactoside α-mannosyltransferases belonging to the GT139 family, were identified. NMR and methylation GC–MS analyses of GXMGal from the *cgm1* (*goe1*) disruptant strain demonstrated the near-complete loss of the galactomannan side chain. The *cgm1* (*goe1*) disruptant also exhibited temperature sensitivity at 37 °C and reduced virulence in mice. Furthermore, the production of interferon gamma (IFN-γ), essential for M1 macrophage activation, was significantly elevated in mice infected with the *cgm1* (*goe1*) disruptant, suggesting that the disruptant is eliminated by the IFN-γ-mediated host immune system. These findings indicate that the GXMGal’s galactomannan side chain is crucial for *C. neoformans*’ stress tolerance at high temperatures and immune evasion.

## Results

### Screening of α-(1 → 4)-mannosyltransferase candidates based on amino acid sequence information

To identify novel α-(1 → 4)-mannosyltransferases, a list of putative Golgi apparatus–localized mannosyltransferase genes was compiled from the genome. According to the CAZy database, 10 putative mannosyltransferase genes are conserved in *C. neoformans* var. *grubii* H99. Specifically, GT15 *KTR3* (CNAG_03832) and GT71 *MNN2* (CNAG_06782) encode α-(1 → 2)-mannosyltransferases (22, 24), whereas GT69 *CMT1* (CNAG_03158), *CAP59* (CNAG_00721), and *CAP6* (CNAG_06016) encode α-(1 → 3)-mannosyltransferases (25, 26). In addition, GT32 *OCH1* (CNAG_00744), *HOC1* (CNAG_05836), *HOC2* (CNAG_01214), *HOC3* (CNAG_00158), and *CSG1* (CNAG_07873) encode α-(1 → 6)-mannosyltransferases or inositol phosphorylceramide α-(1 → 2)-mannosyltransferases (22, 24, 27). Given that no other glycosyltransferase (GT) family with mannosyltransferases was identified, we focused on GTnc, an unclassified GT. Four GTnc genes (CNAG_00129, CNAG_01513, CNAG_02199, and CNAG_04449) were present in the genome, suggesting that the α-(1 → 4)-mannosyltransferase gene could be among them. Each complementary DNA (cDNA) was cloned into pET15-SmaI or pET50b-Amp plasmids and heterologously expressed using *Escherichia coli*. The results showed that CNAG_00129 and CNAG_02199 were successfully expressed as soluble proteins (Figs. S1 and S2*A*), whereas CNAG_01513 and CNAG_04449 were insoluble.

### Glycosyltransferase activities of CNAG_02199 and Nus-tagged CNAG_00129 *in vitro*

**Figure 2:**
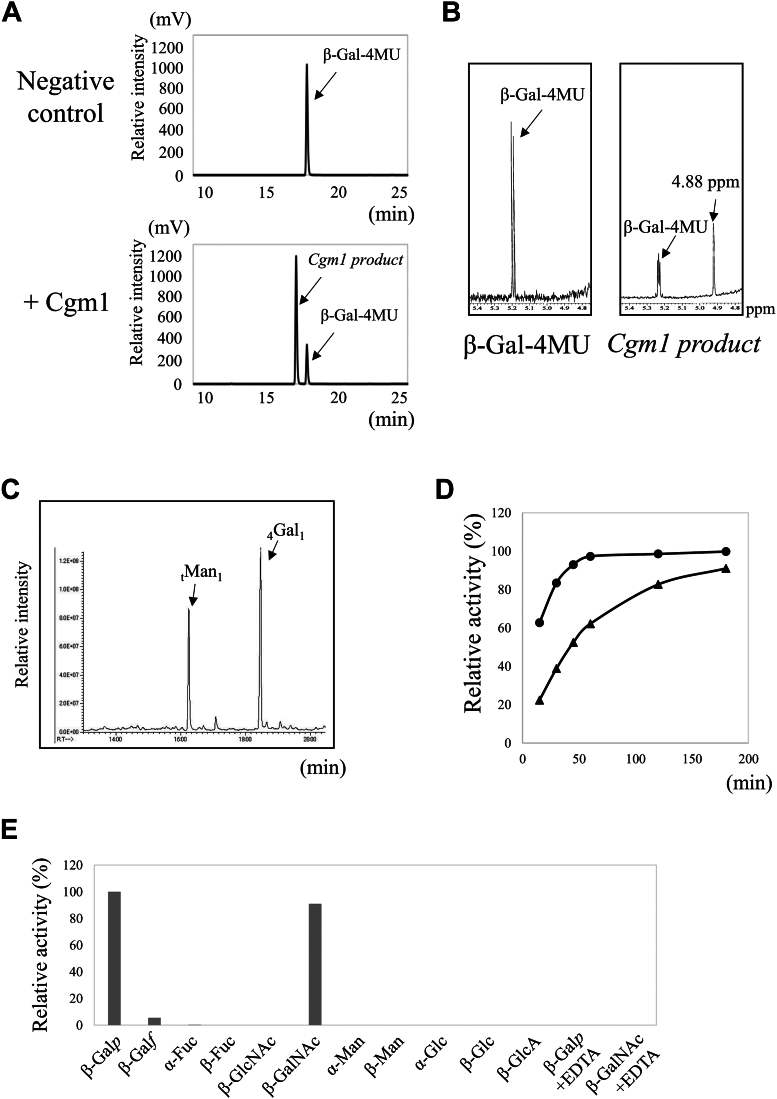
**Measurement of the glycosyltransferase activities of Cgm1.***A*, chromatograms of *in vitro* Cgm1 mannosyltransferase activity assays using 4-methylumbelliferone β-d-galactopyranoside (β-Gal-4MU) as the acceptor substrate. A reaction mixture (40 μl) containing 25 mM Hepes–NaOH (pH 6.8), 50 mM NaCl, 15 mM KCl, 2.5% glycerol, 0.5 mM MnCl_2_, 1.5 mM β-Gal-4MU, 5 mM GDP-Man (donor substrate), and 4 μg of purified Cgm1 was incubated at 30 °C for 3 h. Chromatograms display assay results without enzyme (negative control, *upper panel*), with Cgm1 (*lower panel*). Assays without enzyme yielded only peaks derived from β-Gal-4MU at 18 min, whereas reactions with Cgm1 appeared as two reaction products (termed *Cgm*1 product) at 17.2 min. *B*, ^1^H-NMR analysis of the *Cgm1* product. The 4.88 ppm signal in the ^1^H-NMR spectra corresponds to H-1 at the C-1 position of the underlined Man residues in Manα-(1 → 4)-Galβ. *C*, methylation GC–MS analysis of the *Cgm1* product. The peak at 16:15 min corresponds to the terminal Man residue, whereas the peak at 18:30 min represents the Gal*p* residue with acetylation at the C-4 position. *D*, comparison of reaction time–dependent activity of Cgm1. A reaction mixture (40 μl) containing 25 mM Hepes–NaOH (pH 6.8), 50 mM NaCl, 15 mM KCl, 2.5% glycerol, 0.5 mM MnCl_2_, 1.5 mM β-Gal-4MU (*circle*) or β-GalNAc-4MU (*triangle*), 5 mM GDP-Man, and 4 μg of purified Cgm1 was incubated at 37 °C. Relative activity was calculated as the ratio of the product peak to the sum of the peak heights of the reaction product and unreacted product. *E*, substrate specificity of Cgm1 for its acceptor substrates. A reaction mixture (40 μl) containing 25 mM Hepes–NaOH (pH 6.8), 50 mM NaCl, 15 mM KCl, 2.5% glycerol, 0.5 mM MnCl_2_, 1.5 mM each 4MU-sugars, 5 mM GDP-Man, 4 μg of purified Cgm1, and with or without 5 mM EDTA was incubated at 37 °C for 16 h. Relative activity was calculated as the ratio of the product peak to the sum of the peak heights of the reaction product and unreacted product. When substrates other than β-Gal-4MU, β-Galf-4MU, α-FUc-4MU, and β-GalNAc-4MU were used, the activity was below the detection limit. β-galactoside mannosyltransferase; Cgm, cryptococcal; Man, mannose.

To assess the *in vitro* glycosyltransferase activity, recombinant CNAG_02199 and Nus-tagged CNAG_00129 were produced in a bacterial expression system. The purified enzymes were reacted with various 4-methylumbelliferylated sugars and nucleotide sugars, followed by separation and detection using HPLC and UV detection. CNAG_02199 exhibited glycosyltransferase activity when 4-methylumbelliferylated β-galactose (4MU-β-Gal) and GDP-mannose (GDP-Man) were used as substrates (Fig. 2*A*). Nus-tagged CNAG_00129 also showed mannosyltransferase activity similar to CNAG_02199 (Fig. S2*B*). Therefore, CNAG_02199 and CNAG_00129 were named Cgm1 and Cgm2, respectively. CNAG_02199 has already been named Goe1 (23); we have referred to it as Cgm1 in this article.

Afterward, structural analysis of Cgm1 products using ^1^H-NMR and methylation GC–MS confirmed the presence of a 4MU-β-Gal chemical shift and an α-Man-derived chemical shift at 4.88 ppm (Fig. 2*B*). Methylation GC–MS also detected terminal Man (_t_Man) and 4-O-substituted Gal (_4_Gal_1_) peaks (Fig. 2*C*), indicating that Cgm1 catalyzes the attachment of α-Man to the C-4 hydroxyl group of 4MU-β-Gal. Thus, Cgm1 is a novel β-galactoside α-(1 → 4)-mannosyltransferase.

### Enzymatic properties of Cgm1 and Cgm2

To study the enzymatic properties of Cgm1, its reaction time-dependent enzyme activity and substrate specificity were evaluated (Fig. 2, *D* and *E*). Cgm1 showed a time-dependent increase in reaction product formation (Fig. 2*D*). Regarding substrate specificity toward sugar nucleotides, Cgm1 was shown to react only with GDP-Man. Upon adding the chelating agent EDTA, the glycosyltransferase activity of Cgm1 was completely lost when GDP-Man was used, suggesting that the coordination of the Mn^2+^ ions is required for this activity (Fig. 2*E*). Furthermore, analysis of the substrate specificity toward the accepted substrates revealed that Cgm1 can react with 4MU-β-Gal and 4MU-β-GalNAc (Fig. 2*E*). Nus-tagged Cgm2, like Cgm1, also showed a time-dependent increase in the generation of reaction products (Fig. S3*B*). However, Cgm2 reacted only with 4MU-β-Gal and not with 4MU-β-GalNAc (Fig. S3*B*). Although the presence of an Nus-tag in Cgm2 might alter its properties, these results indicate that Cgm1 and Cgm2 might have slightly different properties.

### Phylogenetic analysis of Cgm homologs

**Figure 3:**
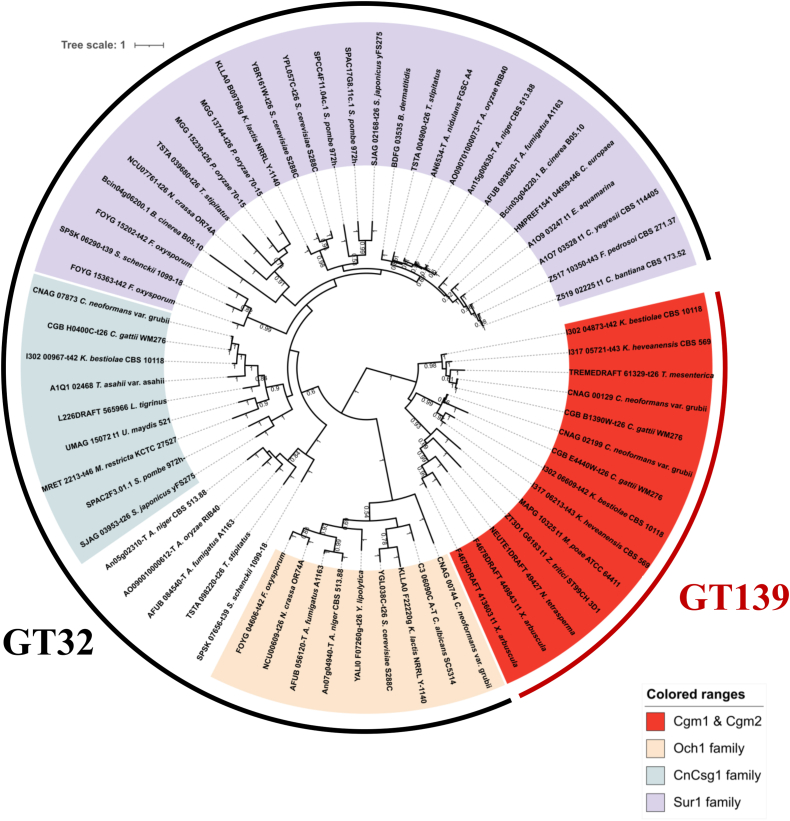
**Phylogenetic analysis of the Cgm homologs and fungal GT32.** Protein sequences were downloaded from FungiDB. Alignment and phylogenetic tree inference were performed using MAFFT and RAxML, respectively, within the ETE v3 framework. The phylogenetic tree was visualized using iTOL. Cgm, cryptococcal β-galactoside mannosyltransferase.

A phylogenetic analysis based on amino acid sequences was performed to determine the evolutionary relationships of Cgm homologs. The identity of the amino acid sequence between Cgm1 and Cgm2 was 39.57%. Next, a database search in FungiDB (https://fungidb.org) revealed that Cgm*-*like glycosyltransferases are found in select fungi but express limited homology to the mannosylinositol phosphoceramide synthase Csg1 and α-(1 → 6)-mannosyltransferase Och1 of the GT32 family. A phylogenetic tree including GT32 glycosyltransferases (Fig. 3) demonstrated that Cgm-like glycosyltransferases form a distinct clade from the known GT32, suggesting that they represent a novel glycosyltransferase family, designated GT139.

### Phenotypic analysis of *cgm1* and *cgm2* disruptants in *C. neoformans* H99

**Figure 4:**
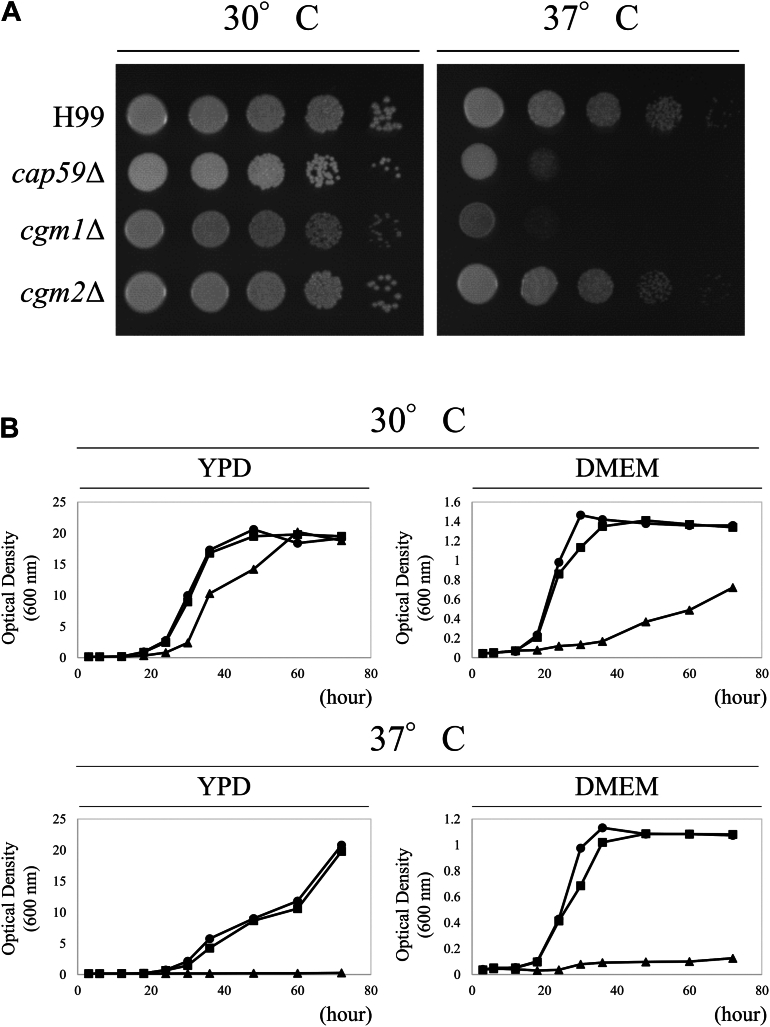
**Growth of *CGM* disruptants.***A*, colony morphology of H99, *cap59*Δ, *cgm1*Δ, and *cgm2*Δ strains on YPD agar at 30 °C and 37 °C for 3 days. The agar medium was inoculated with 10-fold serial dilutions of cells adjusted to 10^6^ cells. *B*, the growth curves of the *Cryptococcus neoformans* strains. The *C. neoformans* H99 (*circles*), *cgm1*Δ (*triangles*), and *cgm1*Δ + *CGM1* (*squares*) strains were precultured in 3 ml of liquid YPD medium at 30 °C and 140 rpm for 16 h. After washing the cells thrice with sterile water, they were counted and inoculated into 100 ml of YPD medium or DMEM supplemented with 25 mM Hepes buffer (pH 7.4) at 1.0 × 10^6^ cells. DMEM, Dulbecco’s modified Eagle’s medium; YPD, yeast extract peptone dextrose.

To evaluate the functions of Cgm1 and Cgm2 *in vivo*, *cgm1* (CNAG_02199) and *cgm2* (CNAG_00129) disruptants were generated in *C. neoformans* H99. The *cap59*Δ strain, a capsular-deficient mutant with growth retardation at 37 °C, served as a control. While *cgm1*Δ exhibited similar growth as the H99 strain at 30 °C, it displayed significant growth retardation at 37 °C, suggesting a temperature-sensitive phenotype (Fig. 4*A*). In contrast, *cgm2*Δ did not exhibit temperature sensitivity, indicating that Cgm1 plays a more critical role in high-temperature survival.

Next, to investigate the detailed growth of *cgm1*Δ, its growth curves were generated in yeast extract peptone dextrose (YPD) and Dulbecco’s modified Eagle’s medium (DMEM) (Fig. 4*B*). The results showed that while *cgm1*Δ exhibited remarkable temperature sensitivity in both culture media at 37 °C, its growth was slightly retarded at 30 °C. Furthermore, a clear growth retardation was observed in DMEM.

### Drug resistance of *cgm1* disruptant

**Figure 5:**
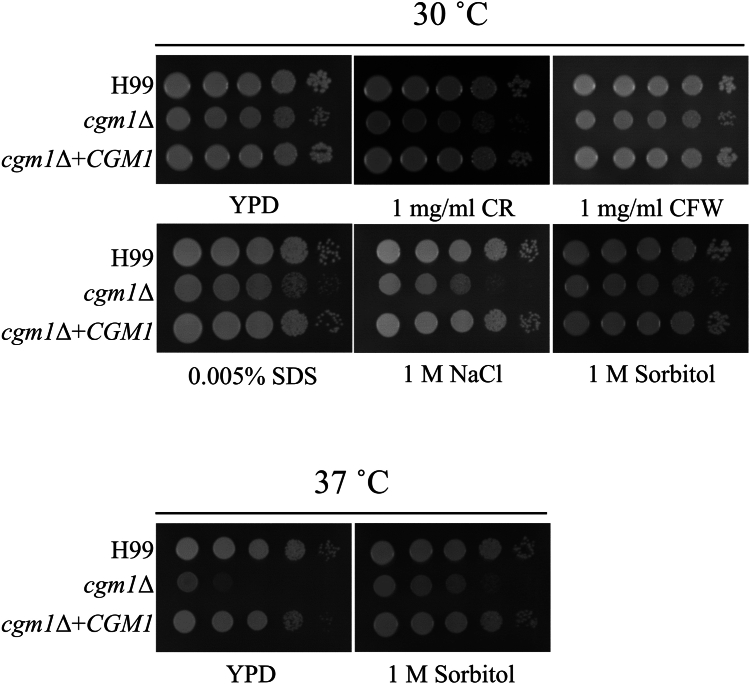
**Drug sensitivity of *cgm1* disruptant.** Colony morphology of H99, *cgm1*Δ, and *cgm1*Δ + *CGM1* on YPD agar supplemented with or without 1 mg/ml Congo red (CR), 1 mg/ml Calcofluor white (CFW), 0.005% SDS, 1 M sorbitol, and 1 M NaCl at 30 °C and 37 °C for 3 days. The agar medium was inoculated with 10-fold serial dilutions of cells adjusted to 10^6^ cells. YPD, yeast extract peptone dextrose.

Growth assays in media containing cell wall stress inducers (Fig. 5) revealed that *cgm1*Δ exhibited slight sensitivity to Congo red, Calcofluor white, or SDS but showed significant sensitivity to 1 M NaCl. However, the sensitivity of *cgm1*Δ toward 1 M sorbitol was not significant, indicating it might be susceptible to high Na^+^ and/or Cl^−^ concentrations. Interestingly, the temperature-sensitive phenotype of *cgm1*Δ was ameliorated by 1 M sorbitol (an osmotic stabilizer), suggesting that Cgm1 is involved in high-temperature stress tolerance through its role in polysaccharide biosynthesis tolerance in *C. neoformans*. These phenotypes, especially temperature sensitive, were also observed in the disruptants of UDP-glucose epimerase gene *uge1*, UDP-galactose transporter gene *ugt1*, and GXMGal β-(1 → 3)-galactosyltransferase gene *ggt2*, which were all GXMGal-deficient mutants, suggesting that Cgm1 is involved in GXMGal biosynthesis.

### Phenotypic analysis of *cgm1* and *cgm2* double disruptants

To understand the physiological role of Cgm2, a double disruptant of *cgm1* and *cgm2* was constructed, and its phenotypes were observed (Fig. S4). The growth of *cgm1*Δ*cgm2*Δ was similar to that of *cgm1*Δ at 30 °C. However, the growth retardation observed for *cgm1*Δ on YPD medium at 37 °C was improved in *cgm1*Δ*cgm2*Δ. In contrast, no improvement was seen in the temperature sensitivity of *cgm1*Δ in DMEM because of the double disruption with *cgm2*.

### Sugar composition and linkage analysis of GXMGal in *cgm1* and *cgm2* disruptants

To determine whether Cgm1 contributes to GXMGal biosynthesis, purified GXMGal fractions from *cap59*Δ, *cap59*Δ*cgm1*Δ, and a *cap59*Δ*cgm2*Δ were prepared.

Methylation GC–MS revealed 15 methylated ester sugars in WT GXMGal, mainly three terminal sugars (Xyl, Man, and Gal), three monosubstituted sugars (3-O-substituted Man, 3-O-substituted Gal, and 6-O-substituted Gal), and three disubstituted sugars (2,3-O-substituted Man, 3,4-O-substituted Gal, and 3,6-O-substituted Gal) (Table 1). Notably, terminal Man, 2,3-O-substituted Man, and 3,4-O-substituted Gal peaks were absent in GXMGal from *cgm1*Δ, whereas the ratios of terminal Xyl and 3-O-substituted Man decreased and the ratios of terminal and 3-O-/6-O-/3,6-O-Gal increased compared with the WT GXMGal. In contrast, GXMGal from *cgm2*Δ showed a composition similar to that of WT GXMGal. These findings suggest that the disruption of *CGM1* alters the galactomannan side chain structure of GXMGal.

**Table 1:** Methylation analysis of GXMGal from the *cgm1* disruptant

Residues^a^	Mol %			
	*cap59*Δ	*cap59*Δ*cgm1*Δ^b^	*cap59*Δ *cgm2*Δ	*cap59*Δ*cgm1*Δ + *CGM1*
tXyl_1_	11.24	ND	8.24	9.16
tMan_1_	11.49	ND	11.88	11.5
tGal*p*_1_	2.26	11.99	3.17	12.59
tGal*f*_1_	5.56	27.75	1.79	2.94
_2_Man_1_	5.21	ND	4.57	1.43
_4_Gal_1_	7.98	ND	9.81	8.52
_3_Man_1_	10.09	ND	9.94	6.32
_6_Man_1_	2.63	ND	2.89	2.37
_3_Gal_1_	15.1	25.83	15.98	9.21
_3,4_Gal_1_	2.73	ND	2.23	1.91
_2,3_Man_1_	8.98	ND	11.74	9.78
_6_Gal_1_	9.32	19.61	12.29	13.68
_2,6_Gal_1_	0.96	6.25	2.16	2.63
_3,6_Gal_1_	2.04	8.56	1.81	6.18

a t means nonreducing terminal.

b ND means none detected.

### ^13^C-NMR analysis of GXMGal in the *cgm1* disruptant

**Figure 6:**
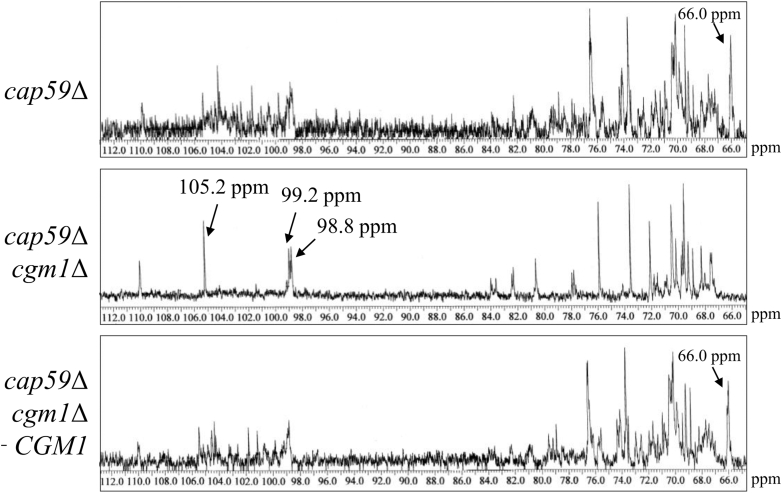
**^13^C-NMR analysis of GXMGal from *cap59*Δ, *cap59*Δ*cgm1*Δ, and *cap59*Δ*cgm1*Δ + *CGM1* strains.** The broad chemical signals at 100.6 to 105.1 ppm in the *cap59*Δ and *cap59*Δ*cgm1*Δ + *CGM1* regions of the ^13^C-NMR spectra originate from the C-1 position of Manα-(1 → 3)-, 2,3-O-substituted-Manα, Xylβ-(1 → 2)-, and Xylβ-(1 → 3)- in the galactomannan side chain. The signals at 66.0 ppm in the *cap59*Δ and *cap59*Δ*cgm1*Δ + *CGM1* of the ^13^C-NMR spectra originate from the C-4 position of Man residues in Manα-(1 → 3)-Manα in the galactomannan side chain. The signals at 98.8, 99.2, and 105.2 ppm in the *cap59*Δ*cgm1*Δ of the ^13^C-NMR spectra originate from the C-1 position of Gal residues, including 3,6-O-substituted Galα, 6-O-substituted Galα, and either unsubstituted Galβ or 3-O-substituted Galβ in α-galactan core and galactomannan side chain. The carbon chemical shifts were referenced to internal acetone at δ 31.07 ppm. GXMGal, glucuronoxylomannogalactan.

To clarify the relationship between Cgm1 and the biosynthesis of the galactomannan side chain, GXMGal was purified and subjected to ^13^C-NMR analysis. In WT GXMGal, a broad chemical shift at 100.6 to 105.1 ppm was detected, possibly derived from the C-1 position of Manα-(1 → 3)-Manα, 2,3-O-substituted-Manα, Xylβ-(1 → 2)-Man, Xylβ-(1 → 3)-Man, and the galactomannan side chain (Fig. 6). Conversely, the GXMGal in *cgm1*Δ exhibited no such chemical shift, whereas a chemical shift at 105.2 ppm, presumably derived from the C-1 position of unsubstituted Galβ or 3-O-substituted Galβ as well as chemical shifts at 99.2 and 98.8 ppm, originating from the C-1 position of 6-O-substituted Galα and 3,6-O-substituted Galα, respectively, were observed. In addition, the 66.0 ppm chemical shift indicative of the C-4 position of Manα-(1 → 3)-Manα in the WT GXMGal was absent in the *cgm1*Δ-derived GXMGal. In contrast, a similar chemical shift was detected in *cgm2*Δ-derived GXMGal as in the WT GXMGal (Fig. S5). These results indicate that *CGM1* disruption causes the loss of the mannosyl residues of the galactomannan side chains in the normal GXMGal.

### Cgm1 activity using mutant GXMGal from the *cgm1*Δ strain as a substrate

**Figure 7:**
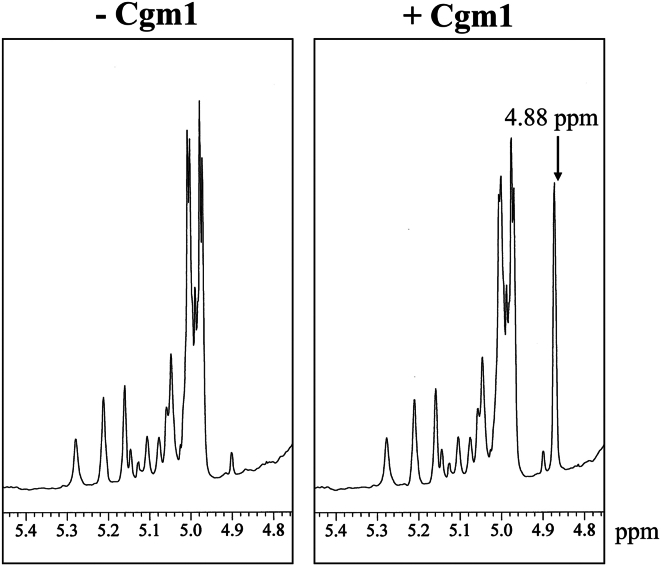
**Mannosyltransferase activity of Cgm1 against GXMGal derived from *cgm1*Δ.**^1^H-NMR analysis of GXMGal derived from *cgm1*Δ. A reaction mixture (4 ml) containing 25 mM Hepes–NaOH (pH 6.8), 50 mM NaCl, 15 mM KCl, 2.5% glycerol, 0.5 mM MnCl_2_, 10 mg of purified GXMGal derived from *cgm1*Δ, 5 mM GDP-Man, and 40 μg of purified Cgm1 was incubated at 37 °C for 16 h. The 4.88 ppm signal in the ^1^H-NMR spectra corresponds to H-1 at the C-1 position of the underlined Man residues in Manα-(1 → 4)-Galβ. GXMGal, glucuronoxylomannogalactan.

To prove that Cgm1 is indeed the GXMGal β-galactoside α-(1 → 4)-mannosyltransferase, we investigated whether Cgm1 exhibits Man residue transfer activity toward GXMGal derived from *cgm1*Δ. Analysis of the reaction products by ^1^H-NMR revealed a chemical shift at 4.88 ppm indicating the presence of Manα-(1 → 4)-Galβ in GXMGal reacted with Cgm1 (Fig. 7). These results strongly indicate that Cgm1 transfers Man residues to the mutant GXMGal derived from *cgm1*Δ.

### Relationship between the elongation of the galactomannan side chains of GXMGal and its pathogenicity in mice

**Figure 8:**
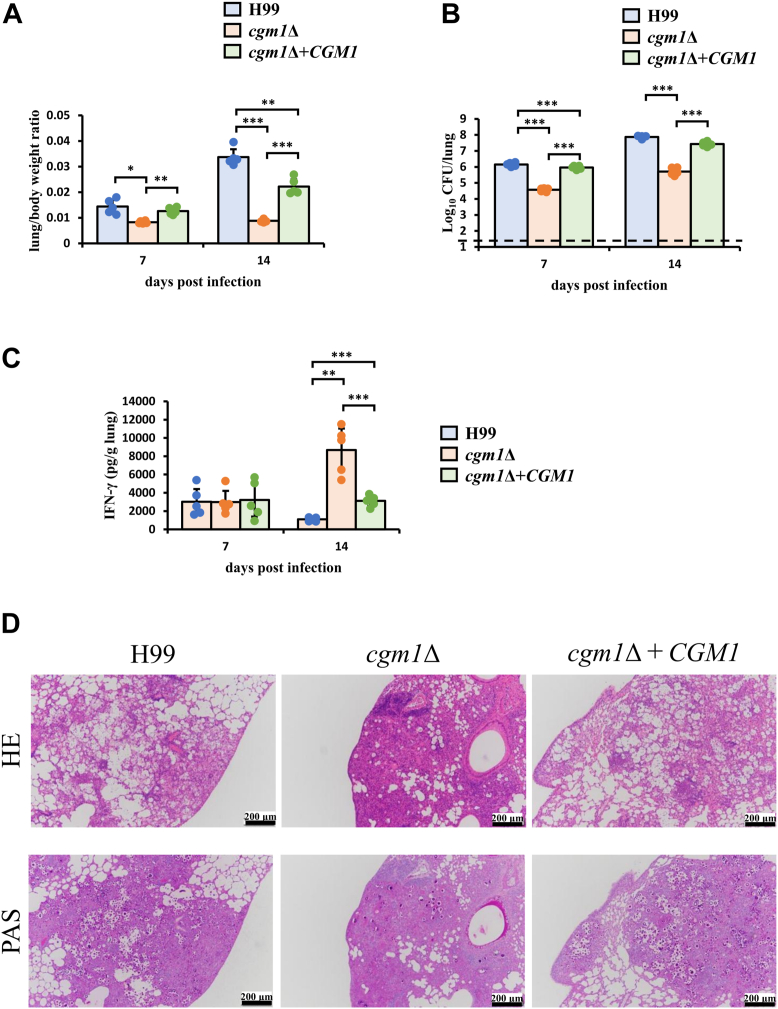
**Effects of *cgm1* disruption in *Cryptococcus neoformans* on mice pathogenicity.** C57BL/6 mice were infected intratracheally with H99 (n = 5), *cgm1*Δ (n = 5), and *cgm1*Δ + *CGM1* (n = 5). *A*, comparison of lung/body weight ratios. *B*, colony-forming units (CFUs) in the lungs on days 7 and 14 postinfection. Each symbol represents an individual mouse; bars indicate mean ± SD. *Asterisks* denote significant differences (∗*p* < 0.05; ∗∗*p* < 0.01; and ∗∗∗*p* < 0.005). The *dashed line* indicates the detection limit of 500 CFU/lung. *C*, comparison of IFN-γ production. Each symbol represents an individual mouse; bars indicate mean ± SD. *Asterisks* denote significant differences (∗*p* < 0.05; ∗∗*p* < 0.01; ∗∗∗*p* < 0.005). *D*, lung sections collected from mice 14 days postinfection with H99, *cgm1*Δ, and *cgm1*Δ + *CGM1* were stained with H&E or PAS and observed under a microscope at 200× magnification. Representative images from five mice are shown. CFU, colony-forming unit. IFN-γ, interferon gamma; PAS, periodic acid-Schiff.

To analyze the effect of galactomannan side chains of GXMGal on pathogenicity, H99, *cgm1*Δ, and *cgm1*Δ complemented with *CGM1* were inoculated into C57BL/6 bloodline mice. The numbers of viable *C. neoformans* cells and cytokine production in the lungs were assessed at 7 and 14 days postinfection. First, lung weight-to-body weight ratios were investigated (Fig. 8*A*). The *cgm1*Δ-infected mice exhibited lower lung/body weight ratios at both time points, indicating reduced growth rates of *cgm1*Δ cells in the lungs compared with the H99-infected mice cohort. To further assess fungal burden, lung homogenates were plated to determine colony-forming units (Fig. 8*B*). The colony-forming units in *cgm1*Δ-infected lungs were approximately 0.1- and 0.01-fold higher than those in H99-infected lungs at days 7 and 14, respectively. Interestingly, IFN-γ production in *cgm1*Δ-infected lungs was approximately 9.0-fold higher than in the WT strain–infected lungs at day 14 postinfection (Fig. 8*C*), suggesting enhanced activation of the immune response, particularly type 1 T-helper (Th1) cells. Conversely, innate immune cell–derived IFN-γ production on day 7 of infection did not differ between strains. Histopathological analysis using H&E and periodic acid-Schiff (PAS) staining was conducted (Fig. 8*D*). PAS staining, which detects sugar chains, showed that H&E-negative, PAS-positive areas corresponded to *C. neoformans* capsular polysaccharides. In the *cgm1*Δ-infected lungs, fewer pathogenic cells were observed, with inflammatory cells forming a granulomatous response around them. These findings suggest that *cgm1*Δ exhibits either delayed growth in the lungs of mice compared with H99 and is more efficiently eliminated by the acquired immunity associated with Th1 cells.

### *In silico* prediction of the Cgm1–substrate complex

AlphaFold3 successfully predicted the complex structure of the Cgm1 protein and its substrate model with relatively high confidence of 95.7, 93.2, and 87.6 for the predicted local distance difference test scores of its catalytic domain, GDP-Man, and methyl-β-galactopyranoside (methyl-β-Galp), respectively (Fig. S6*A*). Cgm1 has a mixed α/β fold consisting of 10 β-strands and 11 α-helices, and the α-helices were predicted to be present around the beta-sheet composed of seven core β-strands. This fold is typical of the GT-A family of glycosyltransferases, which includes the GT32 family (28). The predicted Cgm1 structure was compared with the 3D structures in the Protein Data Bank using the DALI server (Table S1). YeGT, an enzyme of the GT32 family from *Yersinia enterocolitica*, was retrieved with the highest score. YeGT is an α-*N*-acetylglucosaminyltransferase, which specifically modifies host Rho family proteins by attaching GlcNAc to tyrosine residues. Comparison of the predicted structure of Cgm1 with the YeGT structure revealed some similarity in the arrangement of the central β-sheet and the five surrounding α-helices. The metal-binding aspartate–any residue–aspartate (DXD) motif is conserved in both enzymes and appears as DID in Cgm1. However, the other loop regions and the C-terminal structure were predicted to be unique to Cgm1 (Fig. S7*A*). In the active center, several residues that appear to be involved in donor substrate recognition (F129, A130, D134, R137, and N186 in YeGT) were also conserved in Cgm1, but the region predicted to be the binding site for the acceptor substrate appeared to be less conserved (Fig. S7*B*). In the Cgm1 complex, H318 was predicted to bind the active center metal ion Mn^2+^ in addition to D195 and D197 of the DXD motif (Fig. S6*B*). The binding of the donor substrate GDP-Man to the active site appeared to be stabilized by the coordination of the diphosphate to Mn^2+^, the guanosine moiety forming van der Waals interactions with the side chains of Y101 and F174, and the mannose moiety forming hydrogen bonds with D179 and R182. The binding pocket of methyl-β-Galp is predicted to be the molecular surface adjacent to the binding site of GDP-Man, and the C1 group of GDP-Man and the C4-hydroxyl group of methyl-β-Galp are 3.1 Å apart, allowing direct interaction (Fig. S6*B*). This arrangement suggests an S_N_i-like retaining mechanism (29, 30), in which the C4-hydroxyl of β-Galp is deprotonated by the β-phosphate oxygen of GDP-Man and attacks the anomeric carbon of GDP-Man from the same side. Glutamate and aspartate residues, such as E255 of *Mus musculus* xyloside xylosyltransferase 1 (30) and E279 of the yeast α1,2-mannosyltransferase Kre2p/Mnt1p (31), are known to contribute to the binding and reactivity of the acceptor substrate in retaining GTs, which are presumed to proceed *via* an S_N_i-like mechanism. Hydrogen bond formation between E217 and the C5/C6-hydroxyl groups of β-Galp was also implied to be important in an orientation suitable for retaining transfer.

## Discussion

This study aimed to identify a novel glycosyltransferase involved in *C. neoformans* capsular polysaccharide biosynthesis. We successfully identified a novel α-(1 → 4)-mannosyltransferase, Cgm1, through enzyme activity screening (Fig. 1). Capsular polysaccharide biosynthesis in *Cryptococcus* spp. is complex, and to date, only one glycosyltransferase, α-Man β-(1 → 2)-xylosyltransferase (Cxt1 and Cxt2), has been definitively linked to this process (17, 18). While a putative β-galactosyltransferase, Ggt2, was recently identified to be involved in GXMGal biosynthesis through a reverse genetic approach, its enzymatic activity remains undetected, leaving its function undetermined (21). Given the important role of capsular polysaccharides in *Cryptococcus* spp. pathogenicity, understanding their biosynthetic mechanisms is crucial. Our findings demonstrate that the novel mannosyltransferase Cgm1 is involved in GXMGal biosynthesis.

Structural analysis of the recombinant Cgm1 reaction products confirmed that Cgm1 is a β-galactoside α-(1 → 4)-mannosyltransferase. Although Cgm1 could utilize β-GalNAc-4MU as a substrate, GalNAc might not be the original substrate *in vivo*, since it is not known to be one of the sugars found in the *C. neoformans* cell wall. Further analysis of GXMGal from a *cgm1*Δ strain revealed that Cgm1 is the sole α-(1 → 4)-mannosyltransferase involved in the biosynthesis of the GXMGal galactomannan side chain (Fig. 6). Loza *et al.* (23) found an interesting phenomenon in *goe1* (*cgm1*) disruptant, where β-(1 → 3)-glucan in the cell wall is drastically reduced. Therefore, it is considered that the glycans biosynthesized by Goe1 (Cgm1) facilitate the crosslinking of β-(1 → 3)-glucan and β-(1 → 6)-glucan. Our findings may indicate a role for GXMGal in cell wall formation, which remains largely unexplored. Conversely, Cgm2 also transfers mannose to β-galactoside, but its *in vivo* role remains unclear. One possibility is that Cgm2 is an enzyme that is rarely expressed in *C. neoformans* and functions as a Cgm1 adjunct. Alternatively, it is possible that Cgm2 may biosynthesize different glycans than Cgm1, as, interestingly, the temperature sensitivity of *cgm1*Δ*cgm2*Δ (Fig. S4) improved at 37 °C in YPD medium. Since this phenomenon was not observed in DMEM, a minimal medium, certain components found specifically in the YPD media might contribute to the growth of *cgm1*Δ*cgm2*Δ. Cgm2 may contribute to the biosynthesis of basidiomycete-type glycosyl inositol phosphoryl ceramides but not GXMGal, as these structures closely resemble the galactomannan side chain of GXMGal; Cxt1 is also involved in GXMGal and glycosyl inositol phosphoryl ceramide biosynthesis (32). Since the original *in vivo* substrate of Cgm2 remains unknown, a detailed analysis of *cgm2*Δ-derived natural substrates should be conducted.

Disrupting *cgm1* significantly reduced *C. neoformans* infectivity (Fig. 8, *A* and *B*). Similarly, mutations in the *CAP* gene, which encodes GXM biosynthetic enzymes, eliminate virulence (5, 6, 7, 8). While studies on the role of GXMGal in virulence are limited, disruption of the *UGE1* or *UGT1* genes reduces virulence and increases *C. neoformans* phagocytosis by macrophages (14, 16). In addition, loss of *cxt1*Δ leads to a 50% reduction in Xyl residues in GXM and complete loss in GXMGal (18). The significance of the Xyl residues in GXM and GXMGal is evident, as *cxt1*Δ reduces *C*. *neoformans* survival in mouse lungs. A previous study by Villena *et al.* (33) reported that GXMGal induces *Fas-L* expression, macrophage apoptosis, nitric oxide induction, and tumor necrosis factor-α secretion. Furthermore, Rocha *et al.* (34) reported that a GXM-deficient *CAP67 C. neoformans* strain triggers neutrophil extracellular traps *via* reactive oxygen species and a peptidylarginine deiminase-4 signaling pathway. GXMGal also activates dendritic cells, induces Th17 cytokine responses, and protects mice from infection upon administration (35). Compared with the WT strains, *cgm1*Δ-infected mice exhibited increased IFN-γ production and reduced fungal burden in the lungs (Fig. 8, *B* and *C*). Th1 cells produce INF-γ during cryptococcal infections that are important for M1 macrophage activation and pathogen elimination *via* nitric oxide (36). In mice infected with the H99 strain, IFN-γ was shown to be produced by natural killer T cells during innate immunity (37), but its production by Th1 cells was inhibited during acquired immunity. Moreover, immunological studies were conducted using the H99-γ strain, which has been genetically modified to produce IFN-γ (38, 39, 40). Therefore, the increased IFN-γ production from Th1 cells in *cgm1*Δ could be an important finding in elucidating the immune evasion mechanisms of the H99 strain. The difference in fungal burden between the WT and mutant strains, despite no difference in IFN-γ production at 7 days postinfection, is thought to be due to the impaired ability of the mutant strain to grow at 37 °C (Fig. 8, *B* and *C*). Future investigations on the IFN-γ production using *cgm1*Δ-derived GXMGal and the efficiency of differentiation in Th1 cells might help clarify the mechanism by which the galactomannan side chain of GXMGal inhibits acquired immunity.

In conclusion, this study characterized the novel GT139, β-galactoside α-(1 → 4)-mannosyltransferase Cgm1 in *C. neoformans*. Cgm1 homologs are restricted to select fungi, including pathogenic basidiomycetous yeasts, and may play crucial roles in capsular polysaccharide biosynthesis (Fig. 3). Given that Cgm1 homologs are absent in mammals and plants, inhibitors of the targeting Cgm1 could serve as novel antifungal therapeutic agents. Our findings are expected to improve our understanding of fungal polysaccharide biosynthesis.

## Experimental procedures

### Strains and growth conditions

The *C. neoformans* strains used in this study are listed in Table S2. The *C. neoformans* var. *grubii* H99 strain was obtained from the Fungal Genetics Stock Center. The yeast strains were cultured in YPD medium containing 2% w/v glucose, 2% w/v peptone, and 1% w/v yeast extract. For capsular polysaccharide production, all strains were grown in 10% Sabouraud broth medium (0.4% w/v glucose, 0.1% w/v peptone, and 0.1% w/v tryptone) supplemented with 50 mM Mops buffer (pH 7.3). DMEM supplemented with 25 mM Hepes buffer (pH 7.4) was used to study the growth rates.

### Construction of pET15-cgm1, pET15-cgm2, pET50b-Amp-cgm1, and pET50b-Amp-cgm2

*C. neoformans* H99 was cultured under capsular induction conditions in 10% Sabouraud’s medium supplemented with 50 mM Mops buffer (pH 7.3) at 30 °C and 140 rpm for 16 h. Cells were collected, disrupted using glass beads, and vortexed. Total RNA was extracted using RNAiso Plus (Takara Bio), and cDNA was synthesized using PrimeScript RT Master Mix (Takara Bio) according to the manufacturer’s instructions. The *CGM1* and *CGM2* genes, excluding introns and transmembrane regions, were amplified by PCR using cDNA from the *C. neoformans* H99 strain as a template. The transmembrane domains and glycosyltransferase domains of Cgm1 and Cgm2 were predicted using TMHMM-2.0 (https://services.healthtech.dtu.dk/services/TMHMM-2.0/) and MOTIF (https://www.genome.jp/tools/motif/), respectively (Fig. S8). The following primer pairs were used: pET15-cgm1-F and pET15-cgm1-R and pET15-cgm2-F and pET15-cgm2-R, respectively (Table S3). The resulting gene fragments were cloned into the SmaI site of either pET15-SmaI (41) or pET50b-Amp (42) using the In-Fusion HD cloning kit. This yielded the plasmids pET15-cgm1, pET15-cgm2, pET50b-Amp-cgm1, and pET50b-Amp-cgm2. These plasmids were subsequently transformed into SHuffle T7 Express (New England Biolabs) harboring pRARE, a codon-replenishing plasmid.

### Protein purification

Expression and purification of His-tagged recombinant enzymes were performed as described previously (42). Briefly, bacteria were grown in 100 ml of LB medium at 37 °C until they reached an absorbance of 0.2 at 600 nm. Expression was induced by adding 1 mM IPTG, and the cultures were incubated at 18 °C for 65 h. Cells were harvested by centrifugation and disrupted by sonication. Nickel–nitrilotriacetic acid agarose (Wako) was added to the supernatant, and His-tagged proteins were purified according to the manufacturer’s protocol.

### Enzyme assays

The artificial acceptor substrate 4-methylumbelliferyl β-d-galactopyranoside (β-Gal-4MU) was purchased from Sigma–Aldrich, Inc. Standard assays were performed using β-Gal-4MU (1.5 mM) as the acceptor, GDP-Man (5 mM) as the donor, and purified recombinant protein (0.2 μg/μl) in a total reaction volume of 40 μl. Reactions were incubated at 30 °C for 16 h and terminated by heating at 99 °C for 5 min. The enzymatic products of Cgm1 and Cgm2 were analyzed by reverse-phase HPLC using an InertSustain C18 (250 × 4.6 mm; GL Science), as previously described by Kadooka *et al.* (43). The β-Gal-4MU derivatives were detected at UV_300_ absorbance.

### Construction of *cgm1* and *cgm2* disruption strains

The *CGM1* and *CGM2* genes were disrupted in *C. neoformans* H99 and *cap59*Δ strains by inserting *NAT* or *HYG* resistance genes using the CRISPR–Cas9 system (44). A gene replacement cassette encompassing a 50-bp homology arm at the 5′ and 3′ ends of *CGM1* or *CGM2* was generated by recombinant PCR using pNAT_mCherry or pHYG_GFP (45) as templates and the primer pairs xxxx-del-F/xxxx-del-R (where “xxxx” represents *CGM1* or *CGM2*; Table S3). For CRISPR-mediated gene editing, the single guide RNA (sgRNA) scaffold, containing a 20-bp target sequence along with the U6 promoter or U6 terminator, was amplified by PCR using pBHM2329 as a template and the primer pairs M13-F*/*xxxx-gRNA-R1 and xxxx-gRNA-F2*/*M13-R. All PCR fragments were introduced into *C. neoformans* H99 and *cap59*Δ strains *via* electroporation using Gene Pulser II (Bio-Rad). This generated *cgm1*Δ, *cgm2*Δ, *cap59*Δ*cgm1*Δ, and *cap59*Δ*cgm2*Δ strains. Transformants were selected on YPD agar plates supplemented with 100 μg/ml nourseothricin sulfate or 200 μg/ml hygromycin B. The introduction of *NAT* or *HYG* into the respective gene loci was confirmed by PCR using the primer pairs xxxx-comf-F and xxxx-comf-R (Fig. S9).

### Construction of *cgm1* and *cgm2* double disruption strain

The *CGM2* genes were disrupted in the *C. neoformans cgm1*Δ strain by inserting *HYG* resistance genes using the CRISPR–Cas9 system (44). A gene replacement cassette encompassing a 50-bp homology arm at the 5′ and 3′ ends of *CGM2* was generated *via* recombinant PCR using pHYG_GFP (45) as the template and cgm2-del-F/cgm2-del-R as the primers. For CRISPR-mediated gene editing, the sgRNA scaffold, containing a 20-bp target sequence along with the U6 promoter/terminator, was PCR amplified using pBHM2329 as the template and M13-F*/*cgm2-gRNA-R1 and cgm2-gRNA-F2*/*M13-R as the primers. All PCR fragments were introduced into *C. neoformans cgm1*Δ strain *via* electroporation using Gene Pulser II (Bio-Rad) to generate the *cgm1*Δ*cgm2*Δ strain. The transformants were selected on YPD agar plates supplemented with 200 μg/ml hygromycin B. The introduction of *HYG* into the respective gene loci was confirmed by PCR using cgm2-comf-F/R as the primer pair (Fig. S9).

### Complementation of the *cgm1* disruption strain with WT *CGM1*

For complementation analysis, a gene replacement cassette was constructed to reintroduce WT *CGM1* into the *cgm1*Δ strain. This cassette included a homology arm at the 5′ end of *CGM1*, the full-length WT *CGM1* gene containing 3′-UTR, a hygromycin B resistance gene (*HYG*), and a homology arm at the 3′ end of *CGM1*. It was generated by recombinant PCR using *C. neoformans* H99 genomic DNA and pHYG_GFP as templates, along with the primer pairs cgm1-comp-1/cgm1-comp-2, cgm1-comp-3/cgm1-comp-4, and cgm1-comp-5/cgm1-comp-6. The resultant DNA fragment was amplified using the primers cgm1-comp-1 and cgm1-comp-6. The sgRNA scaffold, containing a 20-bp target sequence and either the U6 promoter or U6 terminator, was amplified by PCR using pBHM2329 as a template and the primer pairs M13-F*/*NAT-gRNA-R1 and NAT-gRNA-F2*/*M13-R. All PCR fragments were introduced into *C. neoformans cgm1*Δ and *cap59*Δ*cgm1*Δ strains *via* electroporation. Transformants were selected on YPD agar plates supplemented with 200 μg/ml hygromycin B. The introduction of *HYG* into the gene locus was confirmed by PCR using the primer pair cgm1-comp-comf-F/cgm1-comp-comf-R (Fig. S10).

### Preparation of the GXMGal fraction

GXMGal was purified as previously described by Rocha *et al.* (34) and Kadooka *et al.* (21). Briefly, *cap59*Δ background strains were cultured in 1000 ml of 10% Sabouraud medium at 30 °C with shaking (160 rpm) for 5 days. The culture supernatant was collected by centrifugation, and an equal volume of phenol:chloroform was added, followed by another centrifugation step. The supernatant was collected and dialyzed overnight at 4 °C using a visking tube (Nihon Medical Science, Inc. The polysaccharides were lyophilized into a powdered form. To isolate the GXMGal fraction, the lyophilized powder was dissolved in a 3% cetyltrimethylammonium bromide solution containing 1% borate (pH 9.5), and the resulting precipitate was collected. The fraction was washed with 75% ethanol, dialyzed in water, and lyophilized.

### Methylation GC–MS and NMR spectroscopy

Glycosidic linkage analysis was performed as previously described by Klutts and Doering (18) and Katafuchi *et al.* (46). NMR experiments were also performed as previously described (18, 21). The proton and carbon chemical shifts were referenced to internal acetone at δ 2.225 and 31.07 ppm, respectively.

### Infection assays in mice

Pathogenicity assays were conducted using a C57BL/6 mouse model, as previously described by Sato *et al.* (47) and Miyahara *et al.* (48). Briefly, the *C. neoformans* strains H99, *cgm1*Δ, and *cgm1*Δ + *CGM1* were incubated on potato dextrose agar (PDA) at 30 °C for 2 days before use. Mice were anesthetized *via* intramuscular injection of 0.3 mg/kg midazolam (Fuji Pharma, Japan) and 0.02 mg/kg medetomidine hydrochloride (Zenyaku Kogyo, Japan), followed by intraperitoneal administration of 15 mg/kg pentobarbital (Abbott Laboratories, North Chicago, IL) prior to yeast cell inoculation. Each mouse was then inoculated intratracheally with 1.0 × 10^4^ *C. neoformans* cells in 50 μl using a 24-gauge catheter (Terumo, Japan). Mice were bred at the Institute for Animal Experimentation, Tohoku University Graduate School of Medicine, as previously reported by Miyahara *et al.* (49). Mice were euthanized *via* cervical dislocation on days 7 and 14 post-infection, followed by dissection and removal.

### Enumeration of the viable *C. neoformans*

The fungal burden in the lungs was assessed as previously reported by Nakamura *et al.* (49), Sato *et al.* (50), Sato Y *et al.* (51), and Sato *et al.* (47). Briefly, the lungs were homogenized in 5 ml of distilled water, passed through a stainless steel mesh at room temperature, diluted, and plated on potato dextrose agar. After incubation at 30 °C, the number of emerging colonies was counted.

### Histological examination

Histopathological lung specimens were prepared as previously described by Yamamoto *et al.* (52) and Sato *et al.* (53). Lung specimens from mice were fixed in 10% neutral buffered formalin, dehydrated, embedded in paraffin, sectioned, and stained with H&E and PAS at the Biomedical Research Core, Animal Pathology Platform of Tohoku University Graduate School of Medicine.

### Cytokine assay

Mice were euthanized *via* cervical dislocation on days 7 and 14 postinfection, dissected, and the lungs were collected. Lung tissues were homogenized in 5 ml of phosphate-buffered saline, and cellular residues were removed using a stainless-steel mesh. IFN-γ levels in lung crush fluid were quantified using an IFN-γ ELISA kit (BioLegend).

### Statistical analysis

Data were analyzed using JMP Pro 17 software (SAS Institute) and are expressed as the mean ± SD. Differences between groups were examined using Welch’s *t* test and Bonferroni correction. A *p* value <0.05 was considered statistically significant.

### Three-dimensional structure prediction of Cgm1

The enzyme–substrate complex structure of Cgm1 was predicted using AlphaFold, version 3.0.0 (54). The AlphaFold software was installed on a local workstation following the procedure described on its official GitHub repository (https://github.com/google-deepmind/alphafold3). In addition to the amino acid sequence of Cgm1, Mn^2+^, GDP-Man, and methyl-β-Galp were used as inputs to predict the enzyme–substrate complex structure. The SMILES representations for GDP-Man and methyl-β-Galp were obtained from the PubChem database. The predicted model was visualized and analyzed using PyMOL (Schrödinger, LLC).

## Data availability

All data supporting the findings of this study are available within the article (including its supporting information files). Source data are provided with this article.

## Supporting information

This article contains supporting information.

## Conflict of interest

The authors declare that they have no conflicts of interest with the contents of this article.
