# Supplementary material for: Cgm1 is a β-galactoside α-(1 → 4)-mannosyltransferase involved in the biosynthesis of capsular glucuronoxylomannogalactan in Cryptococcus neoformans
Source: J Biol Chem. 2025 Aug 26;301(10):110632. doi: 10.1016/j.jbc.2025.110632 (PMC12493134; doi:10.1016/j.jbc.2025.110632)
Supplement: Supplemental Figures [file mmc1.pptx]

## Slide 1
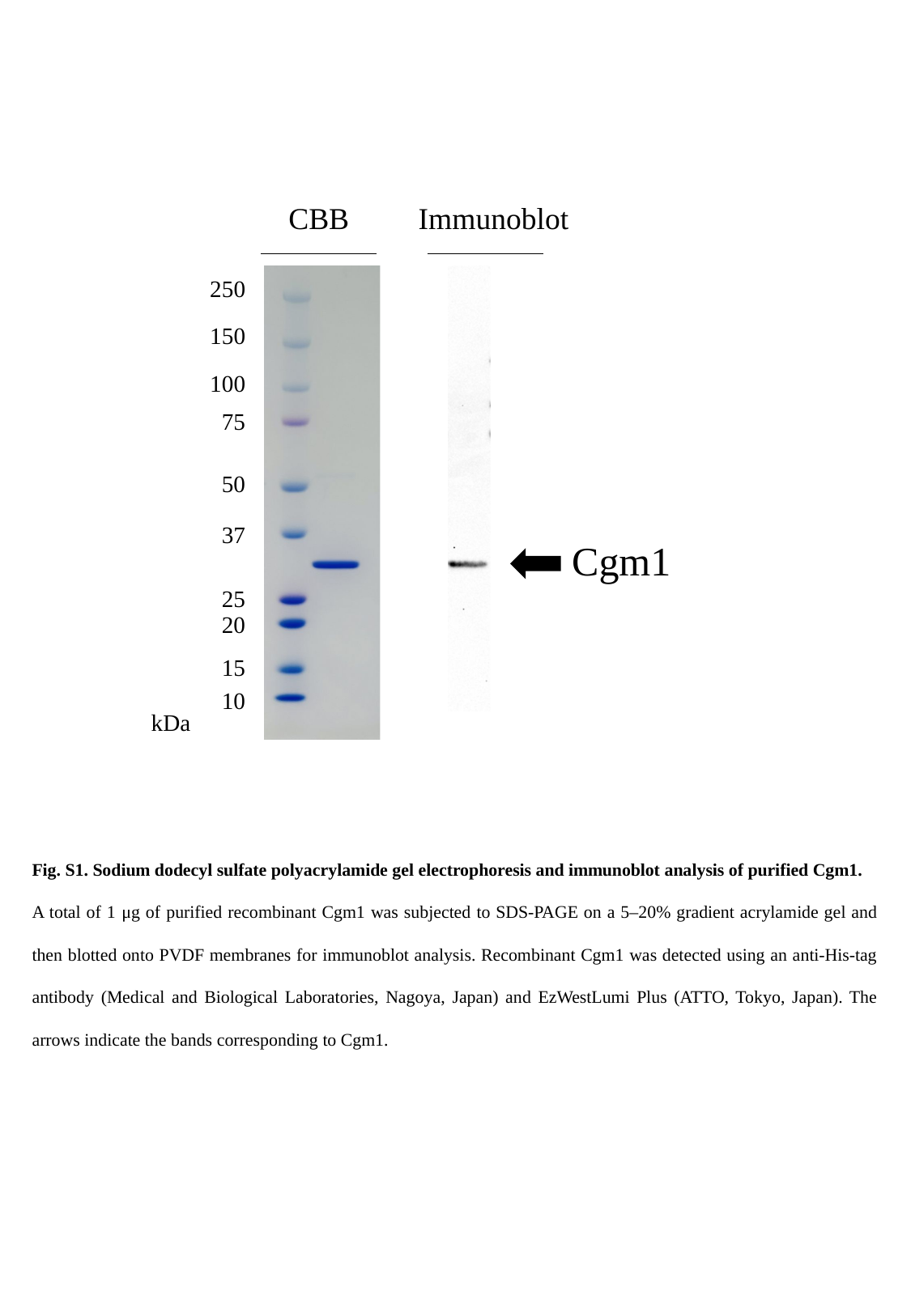

CBB
Immunoblot
250
150
100
75
50
37
Cgm1
25
20
15
10
kDa
Fig. S1. Sodium dodecyl sulfate polyacrylamide gel electrophoresis and immunoblot analysis of purified Cgm1.
A total of 1 μg of purified recombinant Cgm1 was subjected to SDS-PAGE on a 5–20% gradient acrylamide gel and then blotted onto PVDF membranes for immunoblot analysis. Recombinant Cgm1 was detected using an anti-His-tag antibody (Medical and Biological Laboratories, Nagoya, Japan) and EzWestLumi Plus (ATTO, Tokyo, Japan). The arrows indicate the bands corresponding to Cgm1.

## Slide 2
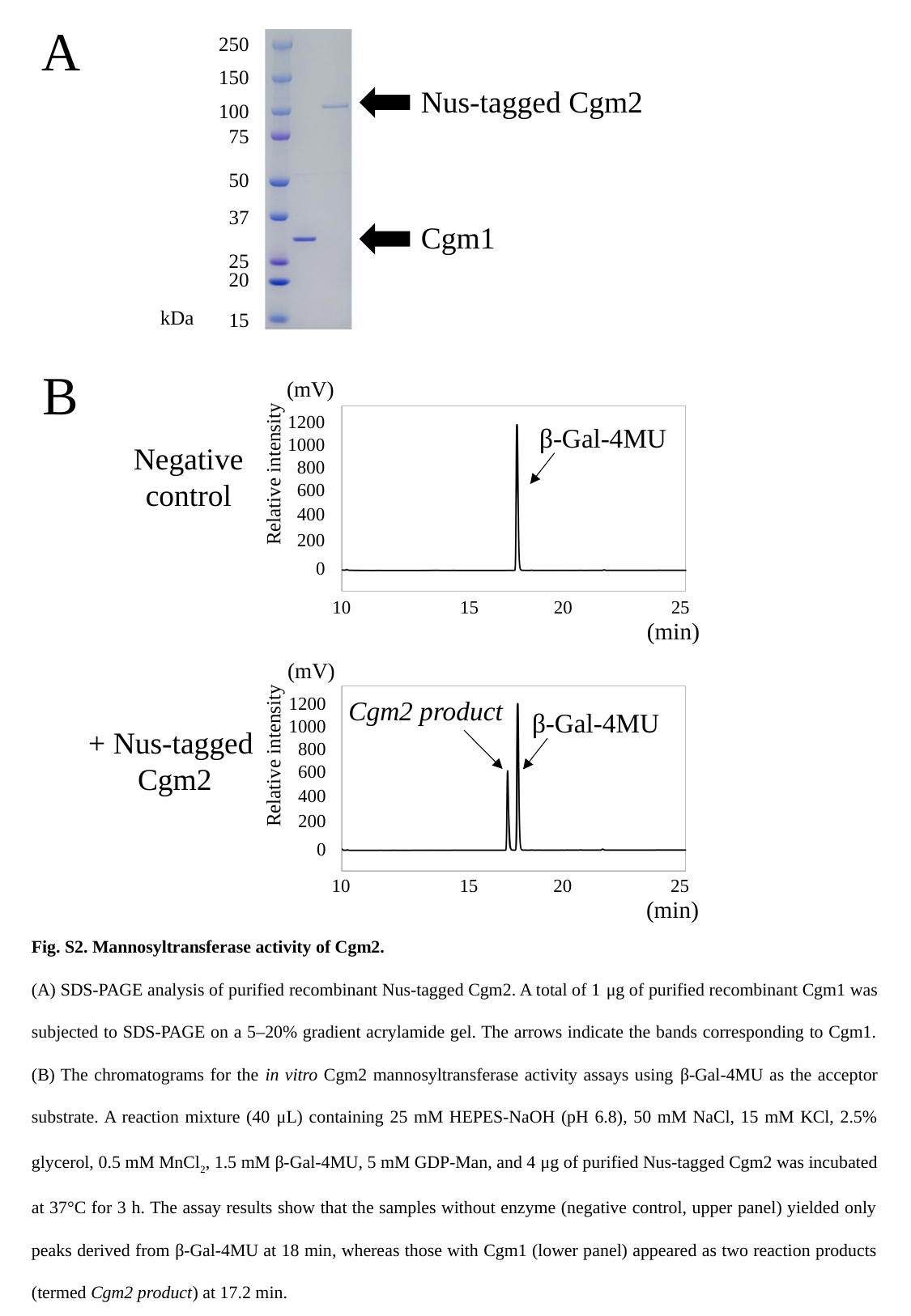

A
250
150
Nus-tagged Cgm2
100
75
50
37
Cgm1
25
20
kDa
15
B
(mV)
1200
β-Gal-4MU
1000
Negative
control
800
Relative intensity
600
400
200
0
10
15
20
25
(min)
(mV)
1200
Cgm2 product
β-Gal-4MU
1000
+ Nus-tagged
Cgm2
800
Relative intensity
600
400
200
0
10
15
20
25
(min)
Fig. S2. Mannosyltransferase activity of Cgm2.
(A) SDS-PAGE analysis of purified recombinant Nus-tagged Cgm2. A total of 1 μg of purified recombinant Cgm1 was subjected to SDS-PAGE on a 5–20% gradient acrylamide gel. The arrows indicate the bands corresponding to Cgm1. (B) The chromatograms for the in vitro Cgm2 mannosyltransferase activity assays using β-Gal-4MU as the acceptor substrate. A reaction mixture (40 μL) containing 25 mM HEPES-NaOH (pH 6.8), 50 mM NaCl, 15 mM KCl, 2.5% glycerol, 0.5 mM MnCl2, 1.5 mM β-Gal-4MU, 5 mM GDP-Man, and 4 μg of purified Nus-tagged Cgm2 was incubated at 37°C for 3 h. The assay results show that the samples without enzyme (negative control, upper panel) yielded only peaks derived from β-Gal-4MU at 18 min, whereas those with Cgm1 (lower panel) appeared as two reaction products (termed Cgm2 product) at 17.2 min.

## Slide 3
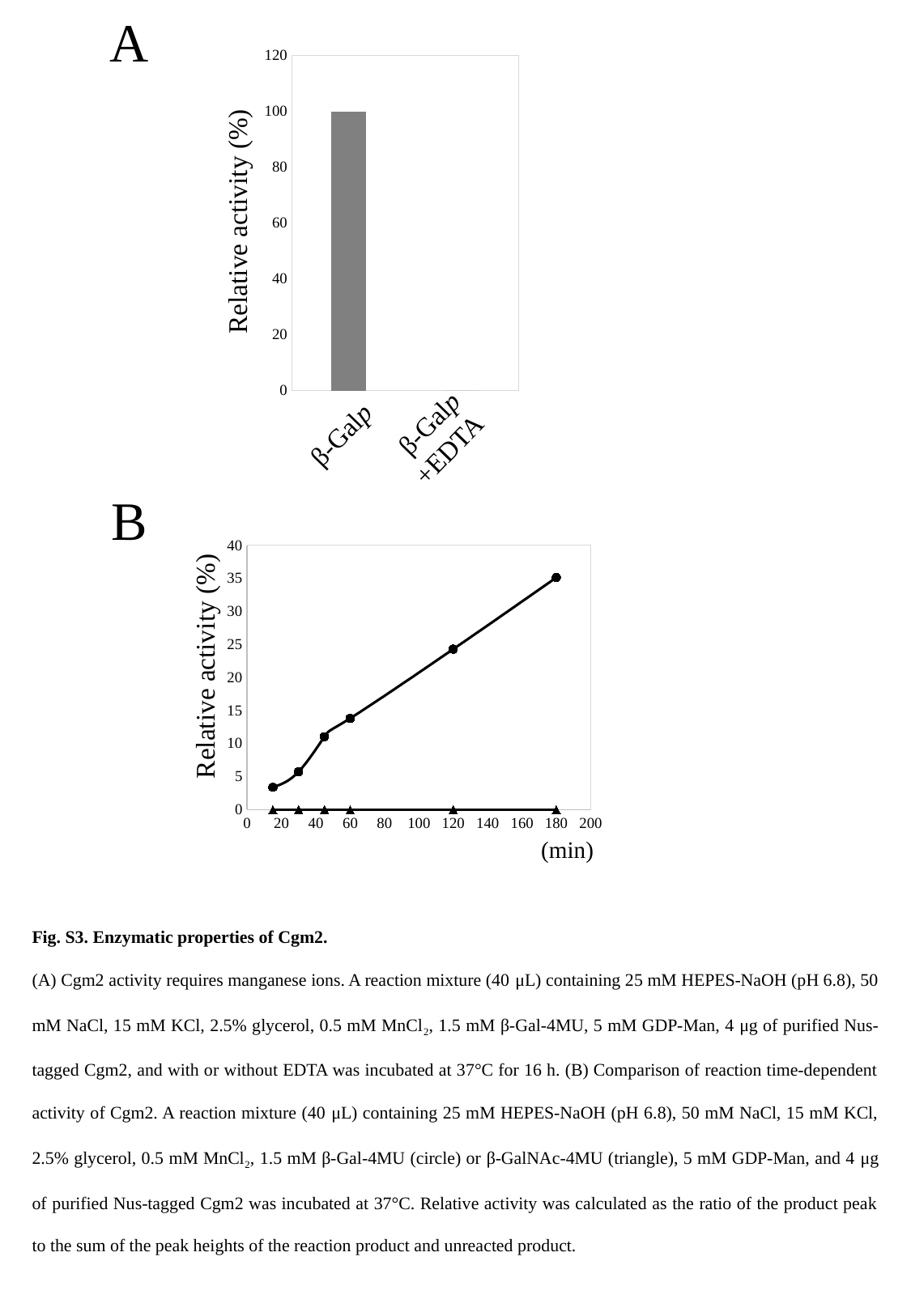

A
### Chart
| Category | |
|---|---|
| a | 100.0 |
| b | 0.0 |Relative activity (%)
β-Galp
+EDTA
β-Galp
B
### Chart
| Category | | |
|---|---|---|Relative activity (%)
(min)
Fig. S3. Enzymatic properties of Cgm2.
(A) Cgm2 activity requires manganese ions. A reaction mixture (40 μL) containing 25 mM HEPES-NaOH (pH 6.8), 50 mM NaCl, 15 mM KCl, 2.5% glycerol, 0.5 mM MnCl2, 1.5 mM β-Gal-4MU, 5 mM GDP-Man, 4 μg of purified Nus-tagged Cgm2, and with or without EDTA was incubated at 37°C for 16 h. (B) Comparison of reaction time-dependent activity of Cgm2. A reaction mixture (40 μL) containing 25 mM HEPES-NaOH (pH 6.8), 50 mM NaCl, 15 mM KCl, 2.5% glycerol, 0.5 mM MnCl2, 1.5 mM β-Gal-4MU (circle) or β-GalNAc-4MU (triangle), 5 mM GDP-Man, and 4 μg of purified Nus-tagged Cgm2 was incubated at 37°C. Relative activity was calculated as the ratio of the product peak to the sum of the peak heights of the reaction product and unreacted product.

## Slide 4
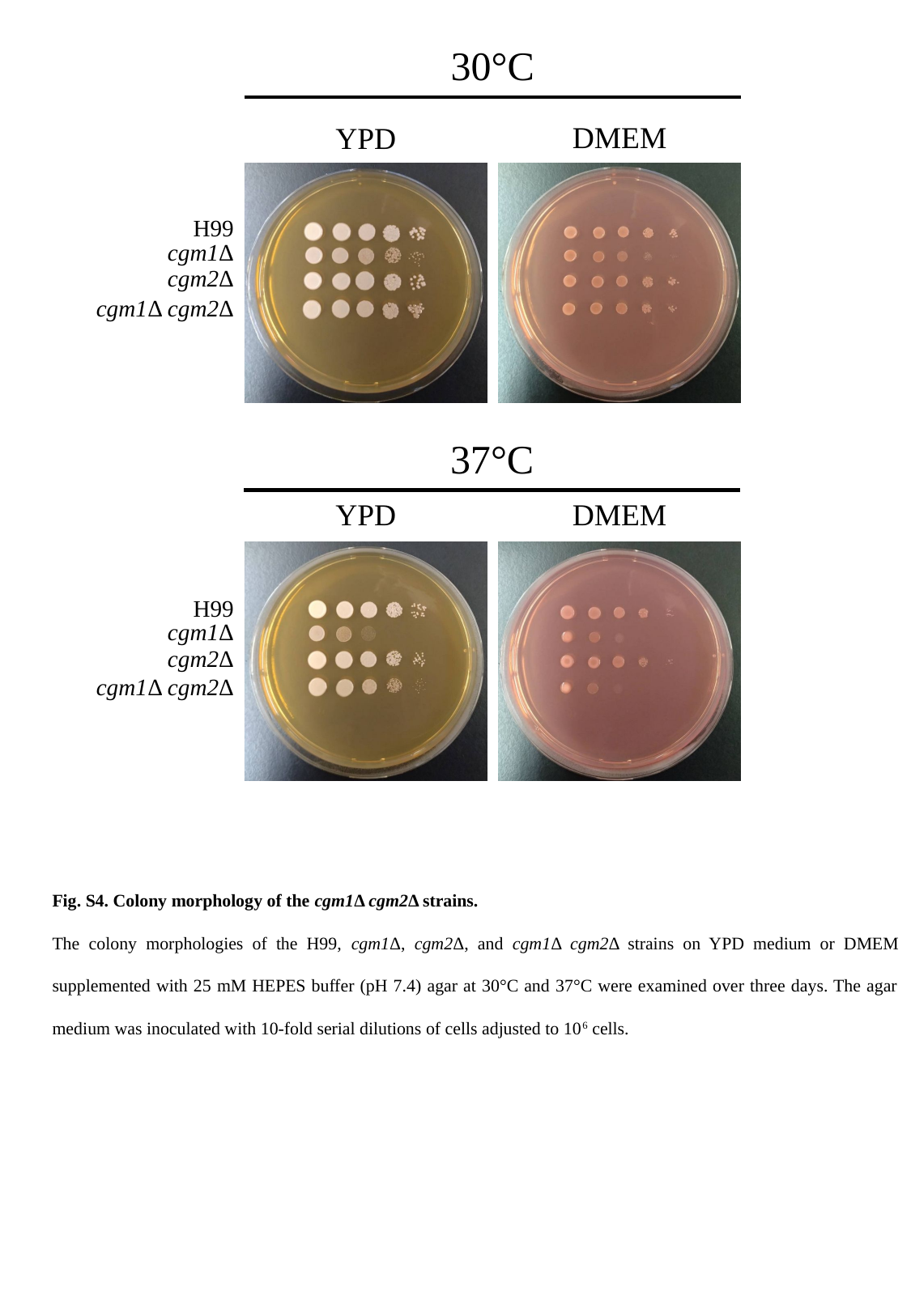

30°C
DMEM
YPD
H99
cgm1Δ
cgm2Δ
cgm1Δ cgm2Δ
37°C
DMEM
YPD
H99
cgm1Δ
cgm2Δ
cgm1Δ cgm2Δ
Fig. S4. Colony morphology of the cgm1Δ cgm2Δ strains.
The colony morphologies of the H99, cgm1Δ, cgm2Δ, and cgm1Δ cgm2Δ strains on YPD medium or DMEM supplemented with 25 mM HEPES buffer (pH 7.4) agar at 30°C and 37°C were examined over three days. The agar medium was inoculated with 10-fold serial dilutions of cells adjusted to 106 cells.

## Slide 5
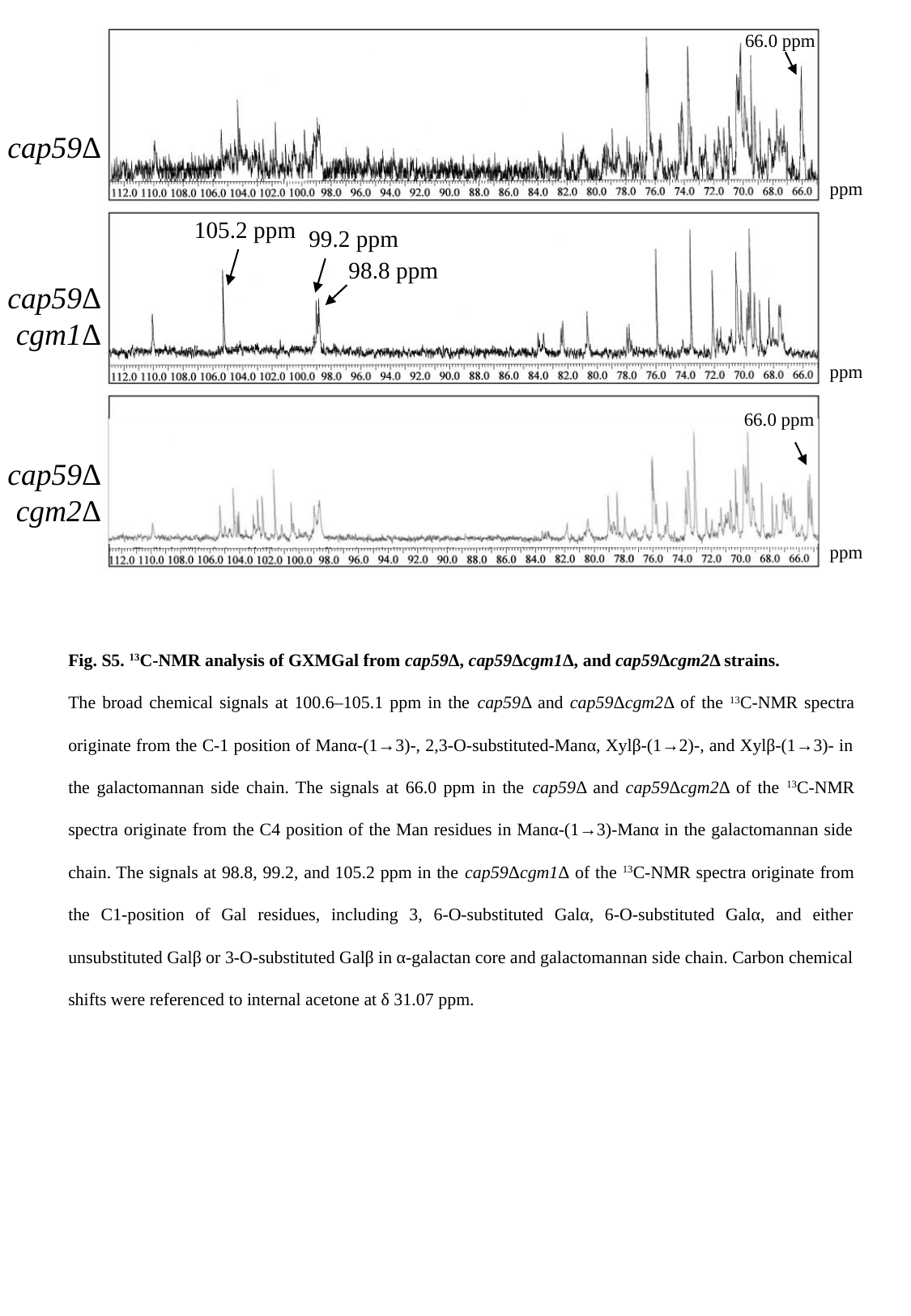

66.0 ppm
cap59Δ
ppm
105.2 ppm
99.2 ppm
98.8 ppm
cap59Δ
cgm1Δ
ppm
66.0 ppm
cap59Δ
cgm2Δ
ppm
Fig. S5. 13C-NMR analysis of GXMGal from cap59Δ, cap59Δcgm1Δ, and cap59Δcgm2Δ strains.
The broad chemical signals at 100.6–105.1 ppm in the cap59Δ and cap59Δcgm2Δ of the 13C-NMR spectra originate from the C-1 position of Manα-(1→3)-, 2,3-O-substituted-Manα, Xylβ-(1→2)-, and Xylβ-(1→3)- in the galactomannan side chain. The signals at 66.0 ppm in the cap59Δ and cap59Δcgm2Δ of the 13C-NMR spectra originate from the C4 position of the Man residues in Manα-(1→3)-Manα in the galactomannan side chain. The signals at 98.8, 99.2, and 105.2 ppm in the cap59Δcgm1Δ of the 13C-NMR spectra originate from the C1-position of Gal residues, including 3, 6-O-substituted Galα, 6-O-substituted Galα, and either unsubstituted Galβ or 3-O-substituted Galβ in α-galactan core and galactomannan side chain. Carbon chemical shifts were referenced to internal acetone at δ 31.07 ppm.

## Slide 6
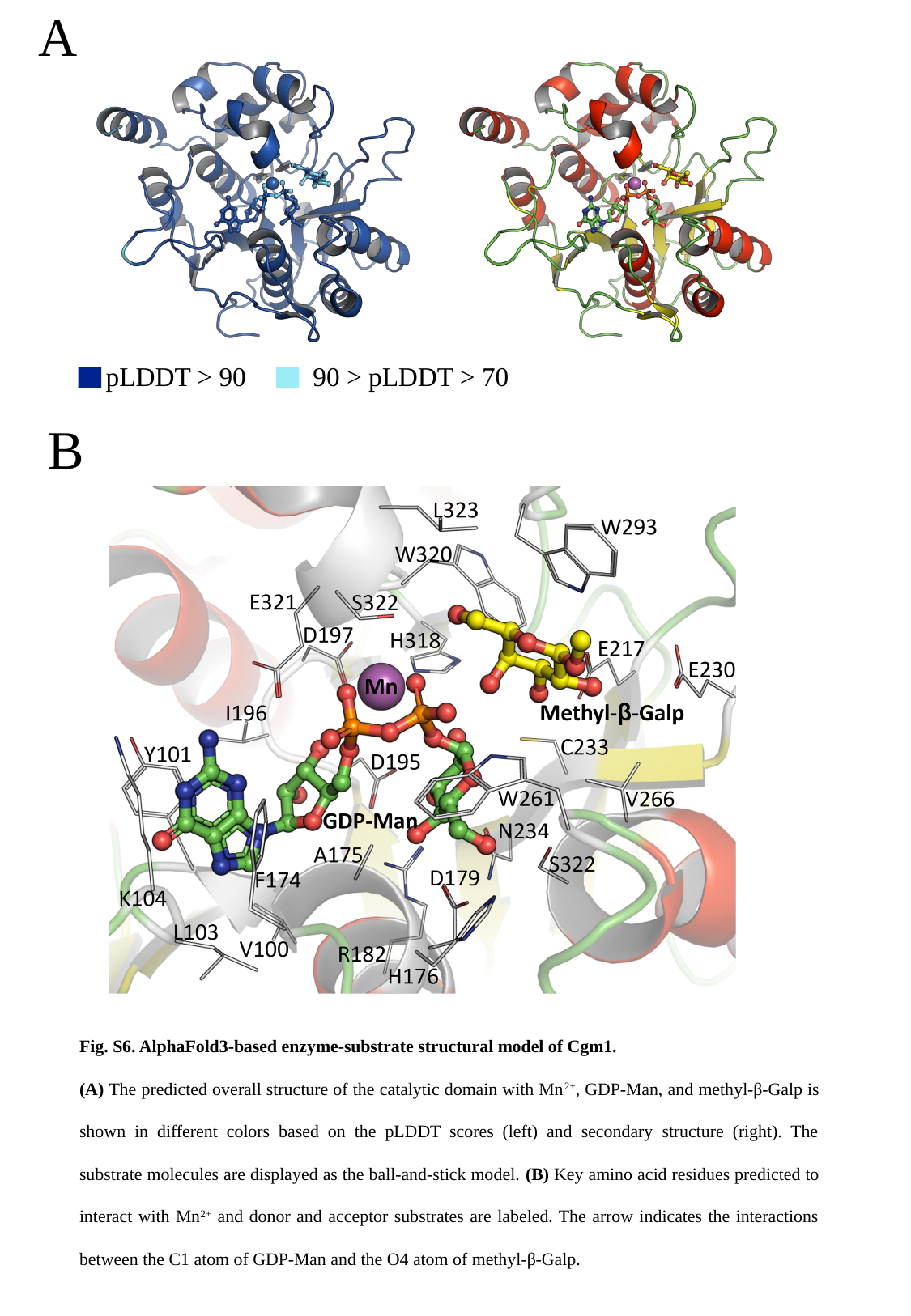

A
pLDDT > 90 90 > pLDDT > 70
B
Fig. S6. AlphaFold3-based enzyme-substrate structural model of Cgm1.
(A) The predicted overall structure of the catalytic domain with Mn2+, GDP-Man, and methyl-β-Galp is shown in different colors based on the pLDDT scores (left) and secondary structure (right). The substrate molecules are displayed as the ball-and-stick model. (B) Key amino acid residues predicted to interact with Mn2+ and donor and acceptor substrates are labeled. The arrow indicates the interactions between the C1 atom of GDP-Man and the O4 atom of methyl-β-Galp.

## Slide 7
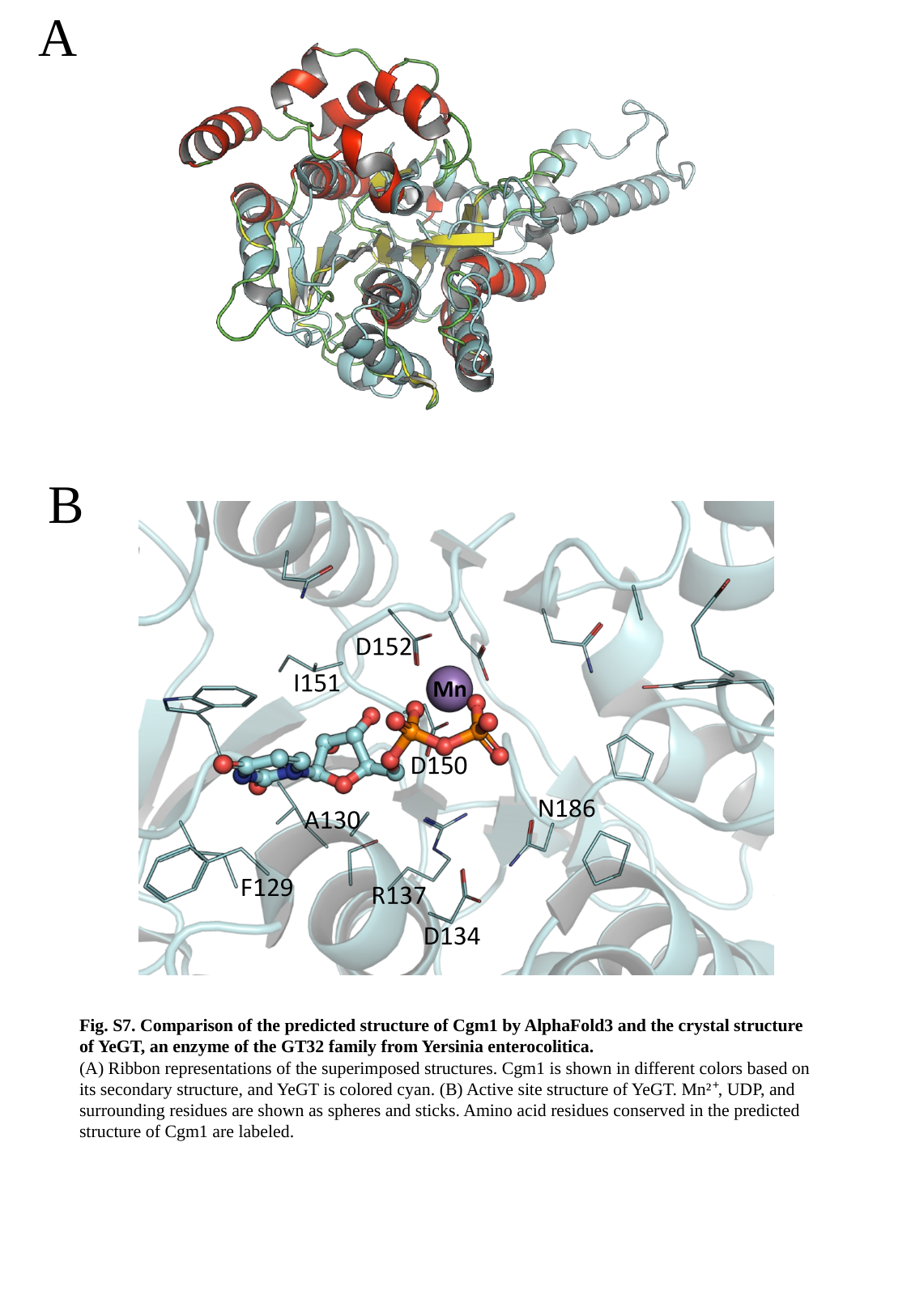

A
B
Fig. S7. Comparison of the predicted structure of Cgm1 by AlphaFold3 and the crystal structure of YeGT, an enzyme of the GT32 family from Yersinia enterocolitica.
(A) Ribbon representations of the superimposed structures. Cgm1 is shown in different colors based on its secondary structure, and YeGT is colored cyan. (B) Active site structure of YeGT. Mn²⁺, UDP, and surrounding residues are shown as spheres and sticks. Amino acid residues conserved in the predicted structure of Cgm1 are labeled.

## Slide 8
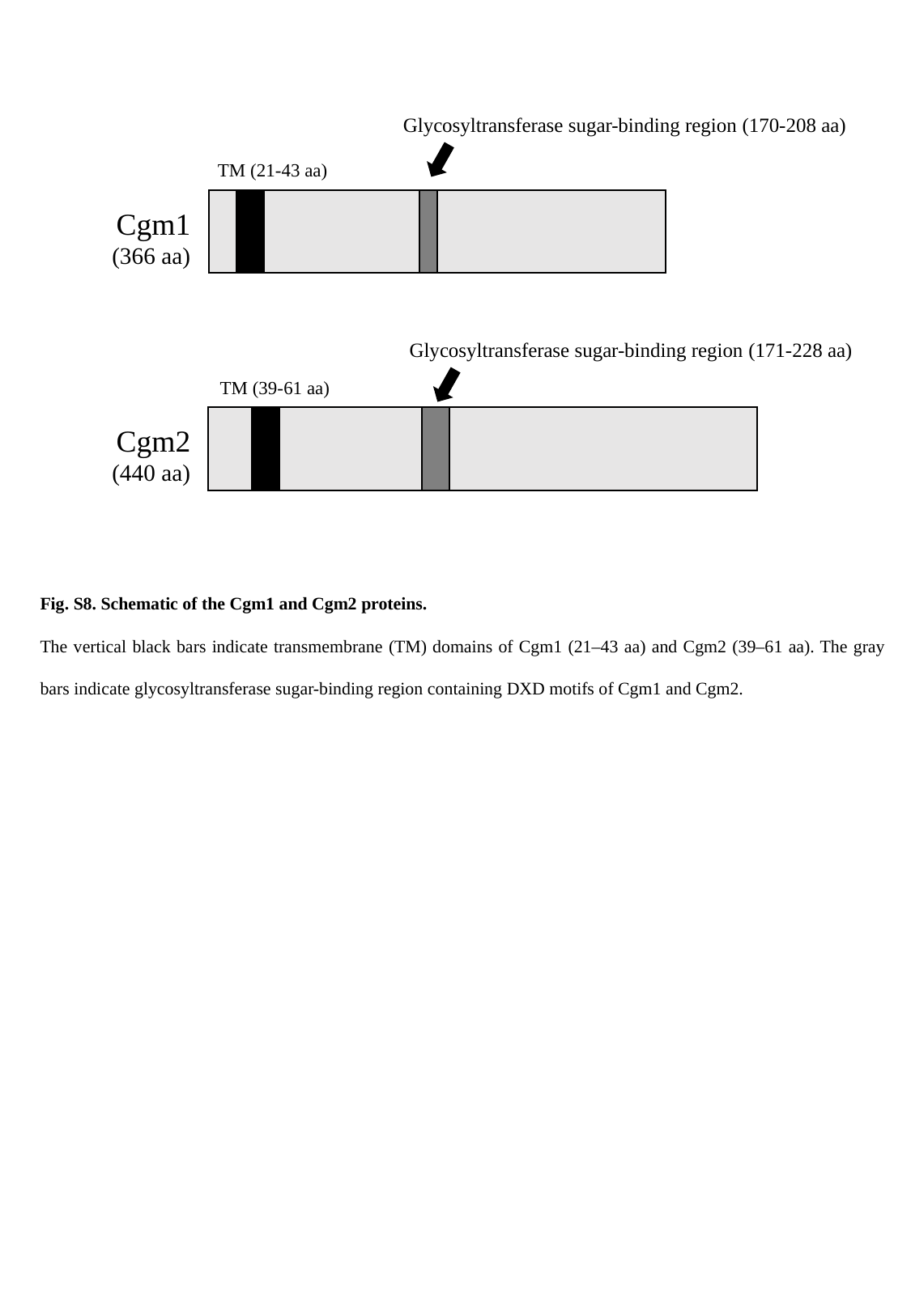

Glycosyltransferase sugar-binding region (170-208 aa)
TM (21-43 aa)
Cgm1
(366 aa)
Glycosyltransferase sugar-binding region (171-228 aa)
TM (39-61 aa)
Cgm2
(440 aa)
Fig. S8. Schematic of the Cgm1 and Cgm2 proteins.
The vertical black bars indicate transmembrane (TM) domains of Cgm1 (21–43 aa) and Cgm2 (39–61 aa). The gray bars indicate glycosyltransferase sugar-binding region containing DXD motifs of Cgm1 and Cgm2.

## Slide 9
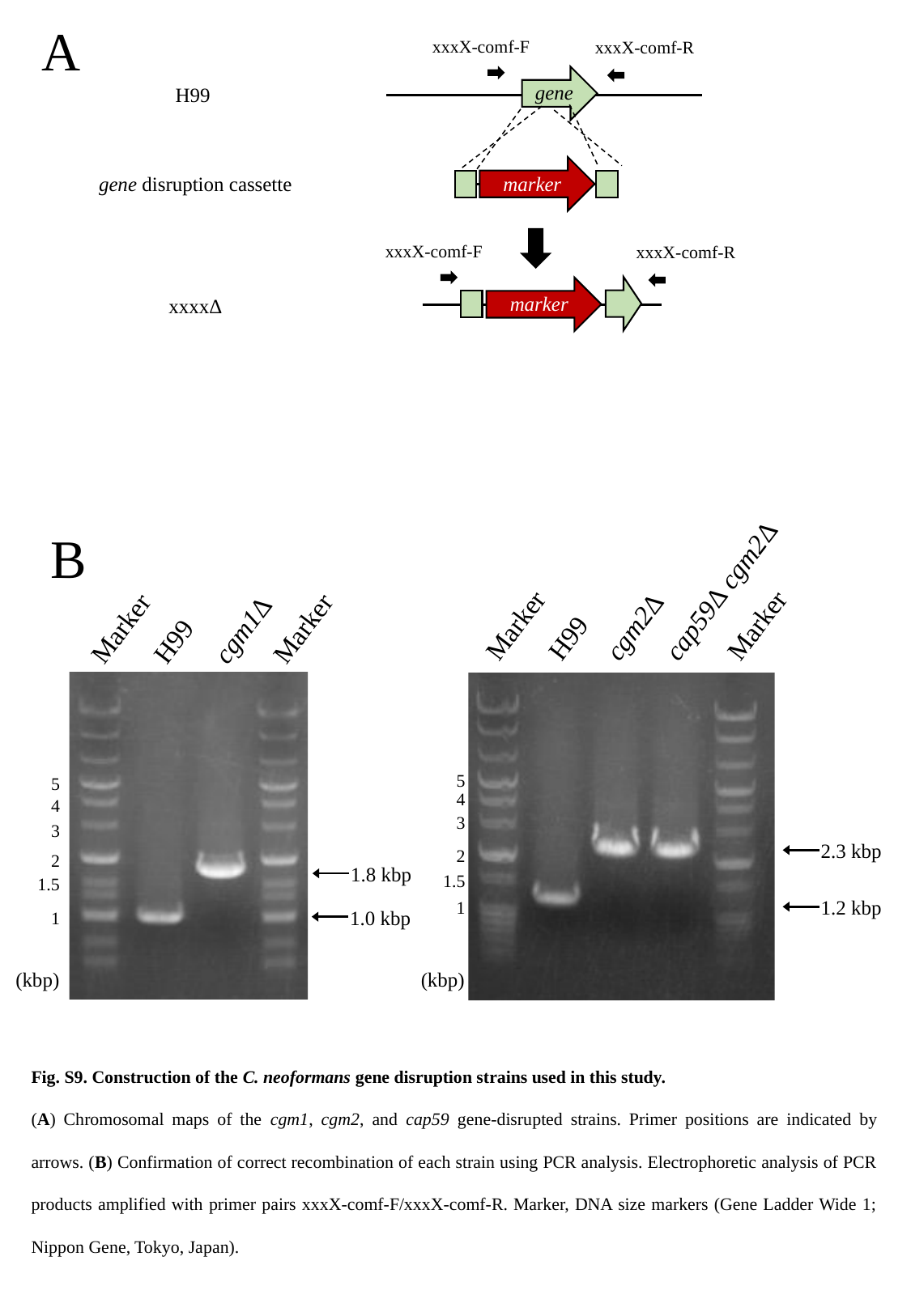

A
xxxX-comf-F
xxxX-comf-R
gene
H99
marker
gene disruption cassette
xxxX-comf-F
xxxX-comf-R
marker
xxxxΔ
B
cap59Δ cgm2Δ
Marker
H99
cgm2Δ
Marker
Marker
H99
cgm1Δ
Marker
5
5
4
4
3
3
2.3 kbp
2
2
1.8 kbp
1.5
1.5
1.2 kbp
1
1.0 kbp
1
(kbp)
(kbp)
Fig. S9. Construction of the C. neoformans gene disruption strains used in this study.
(A) Chromosomal maps of the cgm1, cgm2, and cap59 gene-disrupted strains. Primer positions are indicated by arrows. (B) Confirmation of correct recombination of each strain using PCR analysis. Electrophoretic analysis of PCR products amplified with primer pairs xxxX-comf-F/xxxX-comf-R. Marker, DNA size markers (Gene Ladder Wide 1; Nippon Gene, Tokyo, Japan).

## Slide 10
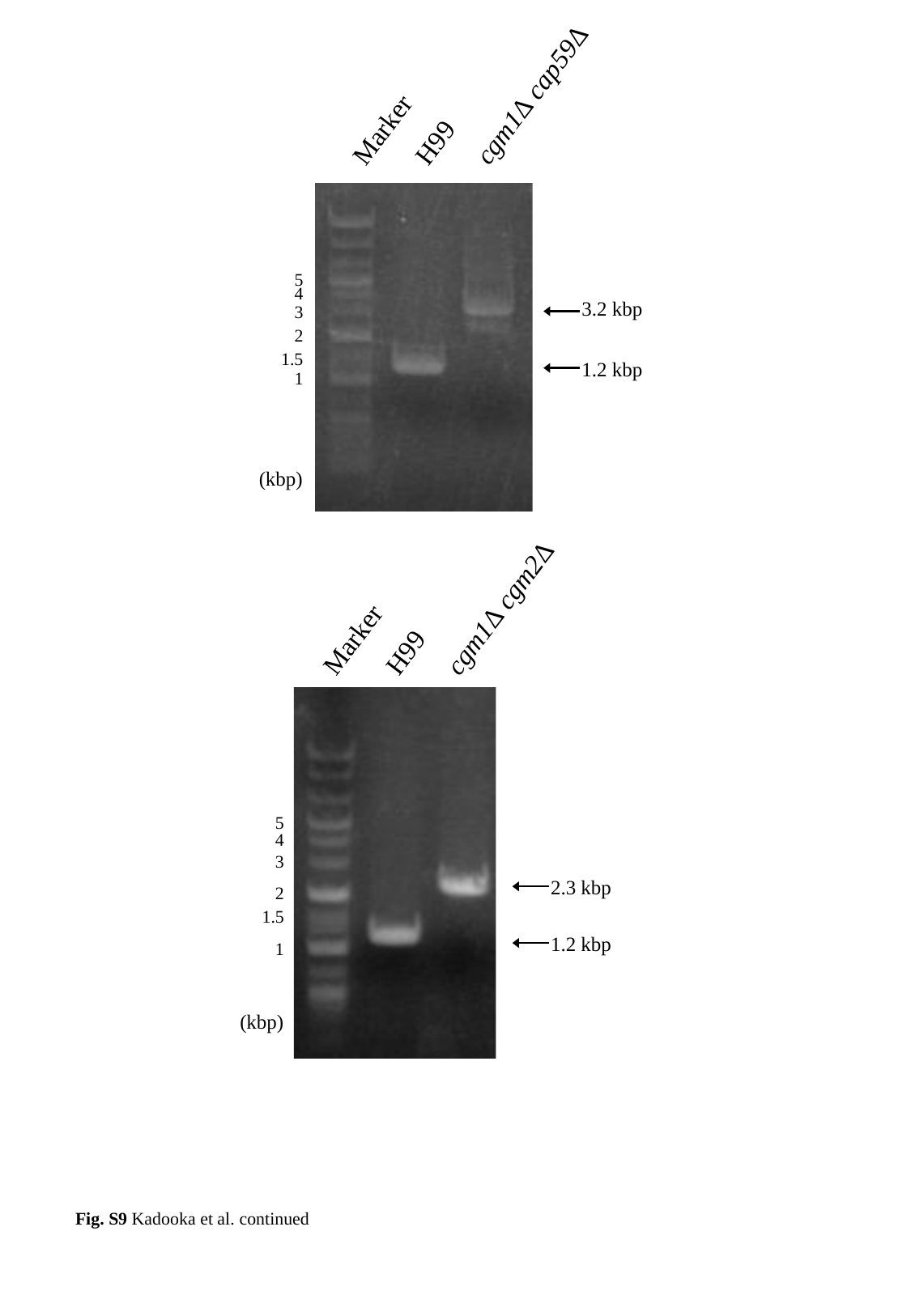

cgm1Δ cap59Δ
Marker
H99
5
4
3.2 kbp
3
2
1.5
1.2 kbp
1
(kbp)
cgm1Δ cgm2Δ
Marker
H99
5
4
3
2.3 kbp
2
1.5
1.2 kbp
1
(kbp)
Fig. S9 Kadooka et al. continued

## Slide 11
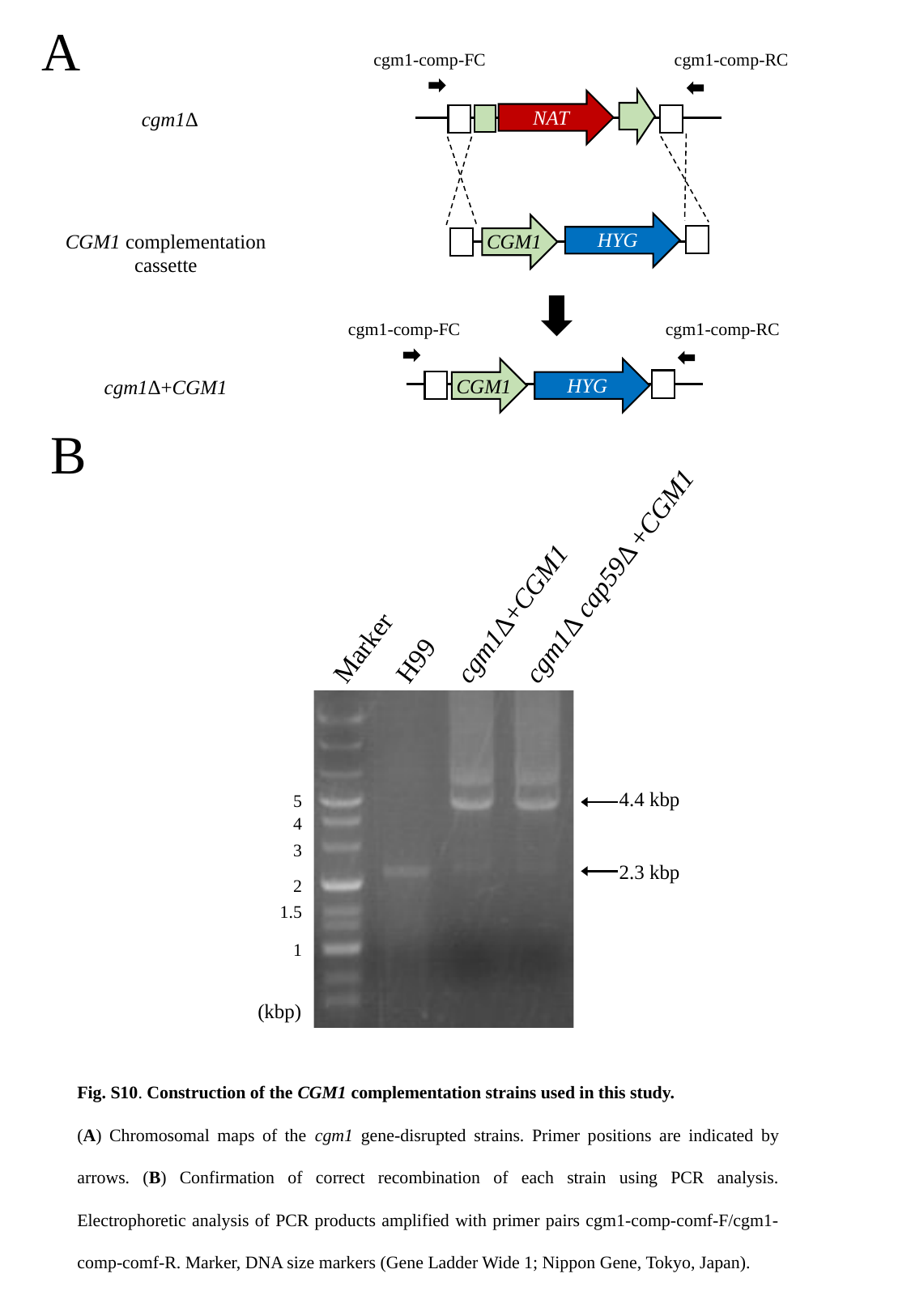

A
cgm1-comp-FC
cgm1-comp-RC
NAT
cgm1Δ
HYG
CGM1 complementation cassette
CGM1
cgm1-comp-FC
cgm1-comp-RC
HYG
CGM1
cgm1Δ+CGM1
B
cgm1Δ cap59Δ +CGM1
cgm1Δ+CGM1
Marker
H99
4.4 kbp
5
4
3
2.3 kbp
2
1.5
1
(kbp)
Fig. S10. Construction of the CGM1 complementation strains used in this study.
(A) Chromosomal maps of the cgm1 gene-disrupted strains. Primer positions are indicated by arrows. (B) Confirmation of correct recombination of each strain using PCR analysis. Electrophoretic analysis of PCR products amplified with primer pairs cgm1-comp-comf-F/cgm1-comp-comf-R. Marker, DNA size markers (Gene Ladder Wide 1; Nippon Gene, Tokyo, Japan).
